# Ultrathin Li Metal Anodes: Quantitative Design Principles and Manufacturability Across Liquid and Solid‐State Batteries

**DOI:** 10.1002/adma.73568

**Published:** 2026-06-01

**Authors:** Cheng Wang, Caoyu Wang, Shuixin Xia, Jodie A. Yuwono, Mingnan Li, Yanqiu Lyu, Ruizhi Zhang, Jun Wang, Jianfeng Mao, Zaiping Guo

**Affiliations:** ^1^ School of Chemical Engineering Adelaide University Adelaide South Australia Australia; ^2^ School of Materials and Chemistry University of Shanghai for Science and Technology Shanghai China; ^3^ Key Laboratory for Liquid−Solid Structural Evolution and Processing of Materials (Ministry of Education) Shandong University Jinan China; ^4^ Department of Materials Science and Engineering City University of Hong Kong Kowloon Hong Kong China

**Keywords:** energy density, interface, Li metal anodes, solid‐state batteries, ultrathin

## Abstract

Li metal anode shows significant potential for advancing high‐energy‐density and commercially viable lithium batteries due to its high specific capacity and low electrochemical potential. However, thinning Li metal encounters serious challenges owing to its mechanical stickiness and fragility during the mechanical rolling process, which severely restricts its practical utilization. Consequently, most current Li metal batteries rely on excessively thick Li foils, leading to substantial resource waste and undermining the pursuit of high energy density. This review highlights the quantitative design principles of ultrathin Li metal (≤15 µm) and elucidates its critical roles in realizing the true potential of Li metal batteries. Emerging strategies for the fabrication of ultrathin Li metal, followed by a critical evaluation of recent advances and persistent challenges in their deployment for both liquid and solid‐state batteries, are summarized. A perspective on future directions for ultrathin Li metal is also presented. Ultrathin Li metal anodes are poised to deliver transformative improvements in energy density, unlocking new opportunities for advanced energy storage systems.

## Graphical abstract

Ultrathin lithium metal anodes (≤15 µm) offer a promising route to high‐energy‐density batteries due to their high capacity and low potential. This review presents design principles for ultrathin Li, evaluates fabrication strategies, and discusses challenges in liquid and solid‐state cells. Future perspectives highlight their potential to enhance energy density and enable next‐generation energy storage technologies.

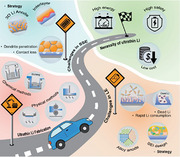

## 1 Introduction

Lithium‐ion batteries (LIBs), featuring high energy density and superior cycling lifespan, outperform other traditional rechargeable batteries such as lead‐acid batteries or nickel‐metal hydride batteries. Since the commercialization of LIBs in 1991, LIBs have rapidly proliferated and are widely used in everyday lives, from consumer electronics to electric vehicles. However, state‐of‐the‐art LIBs based on intercalation chemistry have approached the energy density limit (∼300 Wh kg^−1^) [1, 2], which can hardly satisfy the ever‐growing demand for higher energy density, targeting 500 Wh kg^−1^ and beyond, especially in the electric vehicle market. Advanced battery chemistries beyond LIBs are urgently needed to be developed.

Many strategies have been pursued to enhance the energy density of lithium batteries, such as Lithium–sulfur (Li–S) batteries [3, 4], Li‐air batteries [5, 6, 7], and solid‐state lithium batteries [8, 9, 10]. Among these approaches, Li metal has been regarded as a key component and an ideal anode choice for lithium batteries due to its ultrahigh specific capacity (3860 mAh g^−1^), low electrochemical potential (−3.04 V vs. the standard hydrogen electrode), and low density (0.59 g cm^−3^) [11, 12, 13]. The earliest Li metal batteries were developed by Moli Energy, using a MoS_2_ cathode [14]. However, the severe formation of Li dendrites, leading to internal short circuits, poses serious safety risks and has long impeded the practical deployment of Li metal anodes. As an alternative, graphite anodes deliver enhanced cycling stability and safety compared to Li metal anodes, which has greatly facilitated the commercialization of lithium batteries. However, the relatively low specific capacity of the graphite anode limits the energy density of the batteries.

To achieve a big leap in energy density of lithium batteries, targeting performances of ≥500 Wh kg^−1^ and ≥1000 Wh L^−1^, the revival of Li metal anode has been considered the most promising route toward next‐generation high‐energy‐density lithium batteries. In the past few decades, intensive efforts have been focused on stabilizing Li metal by mitigating Li dendrite growth, improving Li plating/stripping Coulombic efficiency (CE), and prolonging cycling lifespan. However, current research on Li metal stabilization primarily focuses on material‐ or anode‐level improvements at the coin cell scale, which uses excessive amounts of Li metal and electrolytes. The effectiveness of these strategies cannot be directly translated to scaled‐up cells, such as pouch cells, which are typically assembled with lean electrolytes and limited Li metal in practical applications or commercial production. To develop an economically viable and truly high specific energy Li metal pouch cell, the amount of Li metal, shown as the negative/positive (N/P) capacity ratio, must be strictly controlled, along with the other technological parameters, including cathode loading and electrolyte volume. The current limitation of Li foil manufacturing—restricted to foils around 50 µm thick, far exceeding the practical requirement—significantly compromises cell energy density and leads to inefficient Li utilization. Therefore, the use of thinner Li metal anodes is highly desirable for achieving greater efficiency in battery manufacturing.

Nevertheless, less attention has been paid to thinning Li metal. Especially, to achieve a volumetric energy density of ≥1000 Wh L^−1^, the permitted Li metal excess should be strictly controlled to less than 17 µm [15]. Herein, fundamental reasons for thinning Li metal from the perspective of electrochemical performance, safety, and cost are systematically reviewed. Notably, achieving uniform ultrathin Li metal remains challenging using conventional extrusion or calendaring methods. Emerging fabrication strategies are highlighted, and the recent advances and practical challenges in Li metal thinning are critically assessed. Finally, the future prospects for the development and application of ultrathin Li metal are discussed.

## 2 The Necessity of Developing Ultrathin Li Metal

Advanced performance and safe electrochemical energy storage technologies, particularly high‐density and low‐cost, have always been a hot topic in both academic research and industrial production. Since Sony pioneered the commercialization of LIBs in 1991, LIBs featuring graphite anodes have rapidly dominated the battery market, driving significant transformations in communication and transportation of modern life. However, LIBs have approached their theoretical energy density limit restrained by the low specific capacity of graphite anode (372 mAh g^−1^), and the electric vehicle market demands a much higher specific energy of ≥ 500 Wh kg^−1^. As a “holy grail” anode, Li metal has been regarded as the ultimate substitute for high‐energy Li batteries. The Li amount, represented by the N/P ratio, is a crucial parameter for determining the overall energy density of cells. However, the currently reported works mostly focused on optimizing the Li metal anode using far excessive amounts of Li. Thick Li metal will inevitably reduce the cell energy density and increase the safety risks. Strict control of Li metal amount is highly desired to minimize energy density sacrifice and enable more practical battery systems.

### 2.1 Energy Density Perspective

**FIGURE 1:**
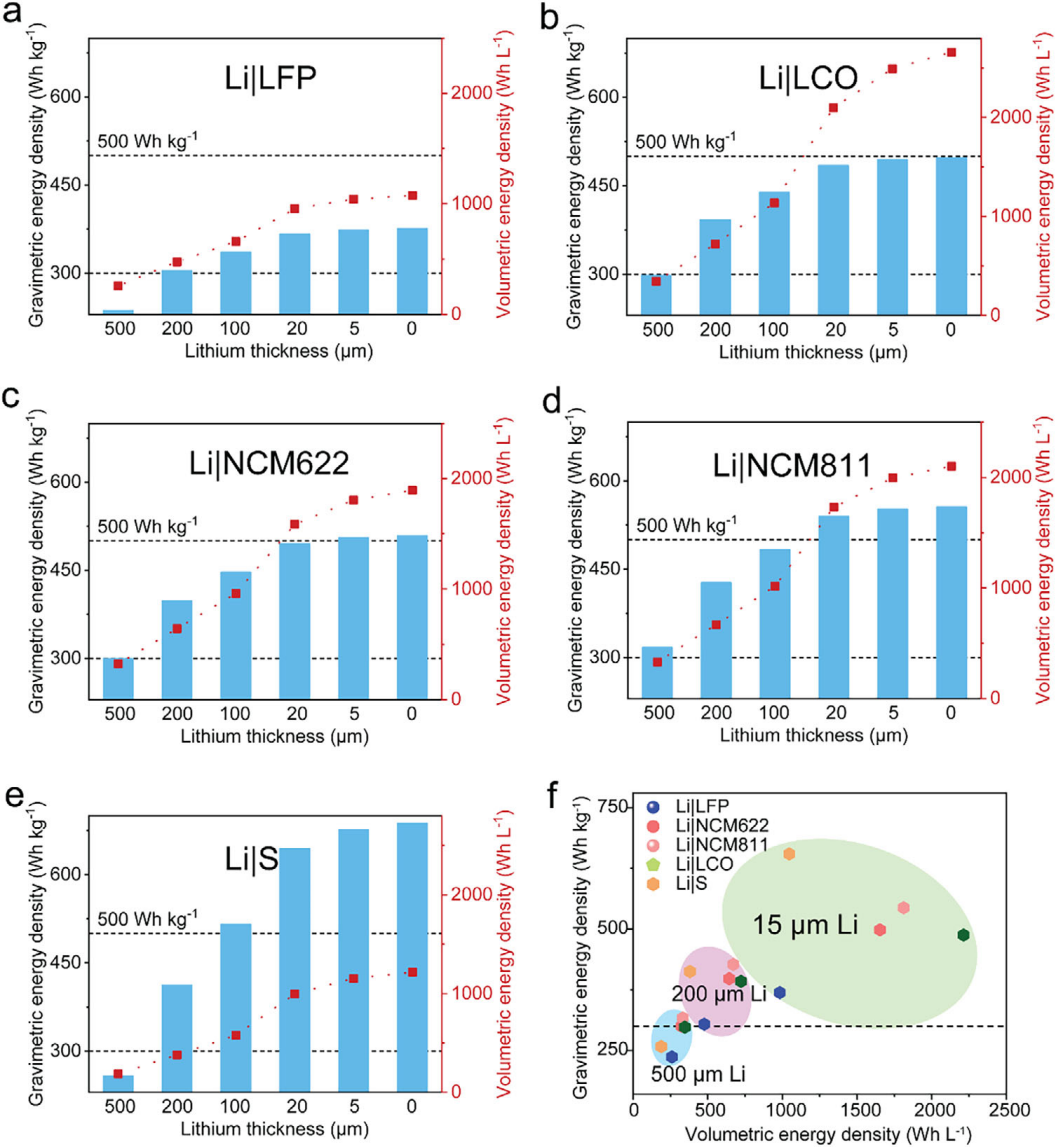
The calculated energy density of a battery system pairing the (a) LFP, (b) LCO, (c) NCM622, (d) NCM811, and (e) S cathode with Li anodes. (f) Comparison of energy density ranges of various cathode materials paired with Li anodes of different thicknesses (500, 200, and 15 µm), illustrating the significant influence of anode thickness on energy density.

Over the past decade, the rapid growth of electric vehicles has imposed increasingly stringent requirements on the energy density of lithium batteries. Among various strategies, coupling Li metal with commercially available or scalable high‐capacity cathode materials represents the most feasible route toward constructing high‐energy systems. However, most current studies on Li metal batteries (LMBs) still rely on a large excess of Li metal. Such a high N/P ratio not only results in substantial lithium resource consumption but also severely compromises the overall cell‐level energy density, which fundamentally contradicts the original motivation for developing LMBs. Figure 1 illustrates the influence of Li metal thickness (varies from 0 to 500 µm) on the gravimetric and volumetric energy densities of LMBs coupled with various cathodes (details of calculations are provided in the Supporting Information). To closely approximate practical applications, cells with LiFePO_4_ (LFP), LiCoO_2_ (LCO), LiNi_0.8_Co_0.1_Mn_0.1_O_2_ (NCM811), LiNi_0.6_Co_0.2_Mn_0.2_O_2_ (NCM622), and S are considered due to their widespread application or extensive investigation. The electrolyte‐to‐capacity ratio is set as 3 g Ah^−1^ to ensure long‐cycle stability in practical use [16].

Among the various cathode materials evaluated, LFP, despite its extensive commercial deployment, delivers a relatively low specific capacity. As a result, achieving a meaningful energy density enhancement (over 300 Wh kg^−1^) over conventional LIBs requires pairing LFP with Li metal anodes thinner than 200 µm (Figure 1a). In comparison, LCO cathodes, which possess a high tapped density (LFP ≈ 2.4 g cm^−3^, LCO ≈ 5 g cm^−3^, NCM811≈ 3.14 g cm^−3^) [16, 17, 18], show a strong dependence of volumetric energy density on Li metal anode thickness. The maximum volumetric performance, approximately 2600 Wh L^−1^, is obtained when the LCO cathode coupled with a 5 µm Li metal anode (Figure 1b). For NCM‐based cathodes, such as NCM622 and NCM811, particularly attractive due to their high specific capacities, elevated discharge voltages, and moderate cost. However, achieving a target gravimetric energy density of 500 Wh kg^−1^ with these cathodes requires the use of Li metal anodes thinner than 20 µm (Figure 1c,d). Among all the systems examined, Li‐S batteries exhibit the highest gravimetric energy density due to the high theoretical specific capacity of S (1675 mAh g^−1^), reaching an ultrahigh value of 687 Wh kg^−1^ for the anode‐free configuration. Notably, even a Li foil thickness of around 100 µm is sufficient for Li‐S batteries to surpass the 500 Wh kg^−1^ threshold, leaving ample room for further improvement through anode thinning (Figure 1e). Figure 1f provides a summary of energy density trends with decreasing Li metal anode thickness across all cathode types, revealing that the adoption of ultrathin Li anodes (≤15 µm) is the prerequisite for achieving gravimetric energy densities greater than 500 Wh kg^−1^ in most systems. Overall, the results reveal that Li metal thickness plays a dominant role in governing both gravimetric and volumetric energy densities of Li metal batteries, regardless of cathode chemistry.

**FIGURE 2:**
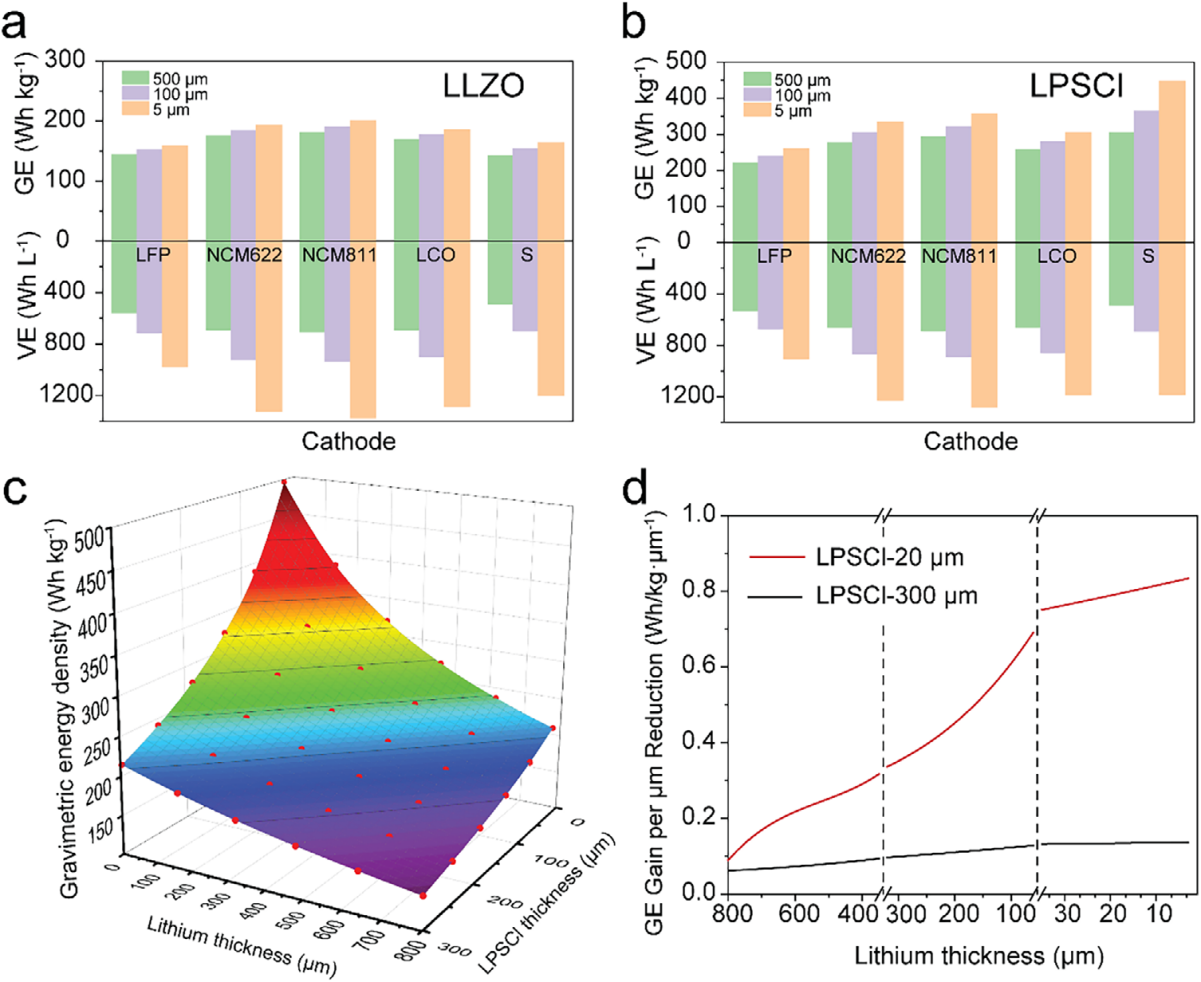
The calculated gravimetric energy density (GE) and volumetric energy density (VE) of ASSLMBs s with (a) LLZO (Li_7_La_3_Zr_2_O_12_) and (b) LPSCl (Li_6_PS_5_Cl). (c) Gravimetric energy density of ASSLMBs as a function of Li anode thickness and LPSCl electrolyte thickness. (d) The calculated energy density gains per micrometer reduction in Li thickness for cells with LPSCl electrolyte thicknesses of 20 and 300 µm.

The thickness of the Li anode is also critical in determining the energy density of all‐solid‐state lithium metal batteries (ASSLMBs). Using the SolidPAC toolkit developed by Oak Ridge National Laboratory, numerical analyses were conducted for representative solid‐state electrolytes (SSEs), including poly(ethylene oxide)‐lithium bis(trifluoromethanesulfonyl)imide (PEO‐LiTFSI), lithium argyrodite (Li_6_PS_5_Cl, LPSCl), lithium lanthanum zirconium oxide (Li_7_La_3_Zr_2_O_12_, LLZO), lithium aluminum titanium phosphate (Li_1.3_Al_0.3_Ti_1.7_(PO_4_)_3_, LATP), and lithium germanium phosphor sulfide (Li_10_GeP_2_S_12_, LGPS). As illustrated in Figure 2a,b, across all cathode types studied, the reduction of Li metal thickness can significantly enhance the energy density of ASSLMBs, particularly when paired with high‐capacity cathodes such as NCM811 and S. For instance, by using LPSCl electrolyte, reducing Li metal thickness from 500 to 5 µm can boost the gravimetric energy density by approximately 25% in Li|NCM811 and 48% in Li|S batteries, respectively.

Currently, the substantial thickness of SSEs contributes most significantly to the total mass and volume of ASSLMBs, often overshadowing the effect of Li anode thickness on energy density. However, with the ongoing development of thinner SSEs, the impact of Li metal thickness will become increasingly critical to achieving further performance improvement. In particular, inorganic SSEs, which generally have large thicknesses (200–300 µm) and high densities, impose severe constraints on volumetric and gravimetric energy performance. Figure 2c,d further elucidate the coupled influence of Li metal and SSE thicknesses on the gravimetric energy density of ASSLMBs. As shown in Figure 2c, the overall energy density is strongly influenced by both Li metal and SSE thicknesses, exhibiting a pronounced nonlinear increase—particularly when both are reduced below ∼100 µm, at which point the energy density surpasses that of conventional LIBs (∼300 Wh kg^−1^). Simultaneously reducing the thickness of Li metal and LPSCl electrolyte to ultrathin levels (<50 µm) enables the energy density exceeding 450 Wh kg^−1^. To quantitatively evaluate how Li thickness contributes under different SSE configurations, Figure 2d presents the energy density gain per micrometer reduction in Li thickness for two representative LPSCl electrolyte thicknesses: 20 and 300 µm. When the LPSCl electrolyte remains thick (300 µm), thinning the Li metal yields only marginal gains (<0.15 Wh kg^−1^ µm^−1^). Whereas, when the LPSCl electrolyte thickness is reduced to 20 µm, the energy density gain becomes highly sensitive to Li thickness, exceeding 0.8 Wh kg^−1^ µm^−1^ once the Li foil is thinned below 15 µm. These results emphasize that in the regime of thin SSEs, the energy benefit of reducing Li thickness is substantially amplified, thus making ultrathin Li anodes a decisive design element for next‐generation ASSLMBs. Therefore, the co‐design of ultrathin Li anodes and thin SSEs is indispensable for unlocking the full energy potential of next‐generation ASSLMBs.

### 2.2 Lithium Resources and Cost Perspective

Lithium, first identified in the mineral petalite by Swedish chemist Johan August Arfwedson in 1817, is relatively scarce in the Earth's continental crust, with an average abundance of approximately 20 ppm [19, 20]. In addition to its geochemical rarity, Li resources are unevenly distributed, with more than 59% of global reserves concentrated in the Li triangle region encompassing Argentina, Bolivia, and Chile [21]. Beyond its critical role in rechargeable batteries, Li compounds are widely consumed in the ceramics, glass, and aluminum industries, which collectively account for approximately 13% of total Li usage (2024) [21]. This multifaceted demand exacerbates supply constraints and intensifies pressure on global Li availability. From an economic perspective, minimizing battery production costs is a fundamental objective to facilitate widespread adoption of electric vehicles. To achieve cost‐competitive with internal combustion engine vehicles, the target cost for electric vehicle battery systems must generally be priced under $100 kWh^−1^ [22, 23]. Extrapolating from commercial LIBs manufacturing, the total cost of cell components should not exceed $10–12 m^−2^. Currently, cathode and current collector materials account for approximately $7 m^−2^, implying that the combined cost of LMAs and separators must be constrained below $5 m^−2^ [24]. However, thick Li metal, often used in laboratory‐scale studies, far exceed this cost threshold due to high material and processing expenses. Consequently, reducing the thickness of Li metal anodes is not only requirements for optimizing materials use, but also for achieving economic feasibility.

### 2.3 Other Advantages of Ultrathin Li Metal Anode

In addition to their primary role as active anode materials, ultrathin Li metal is also employed in the prelithiation of high‐capacity anodes such as silicon (Si) and carbon (C), where precise Li compensation is essential [25, 26]. However, conventional thick Li metal is difficult to process and uniformly distribute, often resulting in uneven prelithiation and localized Li excess. Ultrahin Li metal, by contrast, enable more precise control over Li dosing, thereby improving prelithiation uniformity and device consistency. Additionally, safety concerns also drive the shift toward thinner Li metal electrodes. The inherent high chemical reactivity of Li metal arises considerable safety hazards, particularly at excessive quantities. Reducing the total Li inventory through the usage of ultrathin Li metal can significantly lower the risk of thermal runaway or combustion during battery failure. In pouch cell configurations, especially for practical high‐energy cells, the limited electrolyte supply allows only trace amounts of liquid to reach the newly exposed Li surfaces, which are rapidly converted into ionically insulating “dry SEI” layers. These resistive interphases form where electrolyte wetting is insufficient, suppressing Li^+^ transport and electrochemical activity. Compared with ultrathin Li electrodes, the random and uneven electrolyte penetration within thick Li anodes further promotes the generation of such “dry SEI regions,” thereby exacerbating interfacial instability and performance degradation [27]. Thinner Li metal anodes can help mitigate these interfacial degradations by reducing Li consumption, electrolyte depletion, and SEI accumulation, thereby preserving electrode integrity and extending cell lifespan. Furthermore, ultrathin Li enhances thermal management by facilitating more uniform heat distribution, reducing thermal gradients, and suppressing dendrite growth, all of which are essential for maintaining long‐term battery performance and safety. Additionally, the implementation of ultrathin Li metal promotes the development of wearable and flexible electronics. Thick Li metals inherently lack flexibility, in which large bending angles often lead to delamination, cracking, or electrode fracture. By reducing the Li thickness to 15 µm with a current collector support, the electrode can reliably endure mechanical stress such as bending, rolling, or compression without structural damage. This mechanical adaptability renders ultrathin Li anodes indispensable for the advancement of next‐generation flexible battery systems.

## 3 Challenges of Utilizing Ultrathin Li Metal

Despite the promise of ultrathin Li metal as an anode material for LMBs, offering significant potential for increased energy density, it also faces a series of formidable challenges. These challenges are associated with stringent interface side reactions with electrolyte, Li dendrite growth, mechanical fragility, and other related issues. Here, we explore these challenges in depth, highlighting the critical barriers that must be surmounted to fully exploit the benefits of ultrathin Li metal anodes.

### 3.1 SEI Formation, Dendrites, and “Dead” Li

Due to the high reactivity of Li metal, a SEI layer is spontaneously generated once contacted with the electrolyte [28, 29, 30]. The properties of the SEI layer play a pivotal role in determining the performance of Li metal anodes. A stable and homogeneous SEI is crucial for maintaining a passivating barrier that suppresses continuous and irreversible electrolyte consumption. However, the native SEI formed on Li metal is usually unstable and heterogeneous, with intriguing inhomogeneous Li nucleation/growth and the following notorious Li dendrite growth [31, 32, 33]. As dendrites grow and eventually fracture, parts of the dendritic Li become electrically isolated from the bulk anode, forming what is known as “dead” Li [28, 34, 35, 36, 37, 38]. The growth of Li dendrite and formation of “dead” Li undermine both the CE and cycling lifespan [39, 40, 41]; as they increase the specific surface area of the Li metal, exacerbating parasitic reactions with the electrolyte and causing formation of thick and highly resistive SEI layer [28].

**FIGURE 3:**
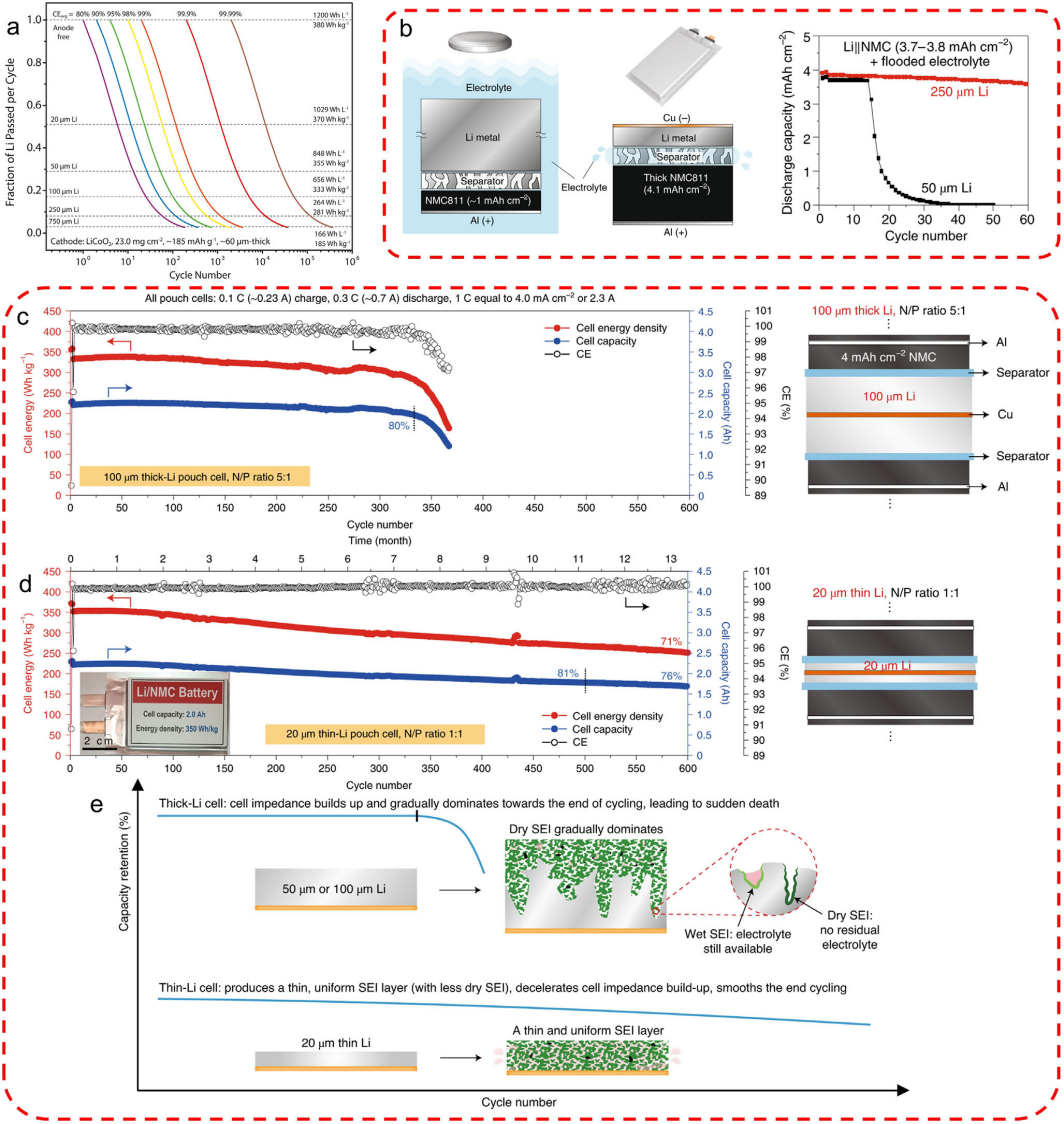
(a) Predicting the energy density and cycle life of lithium metal batteries with varying Li thicknesses. (b) Thick‐Li coin cells exhibit better cycling stability than thin‐Li practical lithium metal batteries. (c) Cycling stability and configuration of 100 µm Li pouch cells. (d) Cycling stability and configuration of 20 µm Li pouch cells. (e) Comparative insights: SEI accumulation and capacity decline in thick Li anodes (50 or 100 µm) vs. SEI optimization and cycle life extension in ultrathin Li anodes (20 µm). (a) Reproduced with permission [18]. Copyright 2020, National Academy of Sciences. (b) Reproduced with permission [16]. Copyright 2019, Springer Nature. (c–e) Reproduced with permission [27]. Copyright 2021, Springer Nature.

For ultrathin Li metal anodes, maintaining long‐term cycling stability is intrinsically far more challenging than for thick Li. From a theoretical perspective, this means that thinner Li requires a much higher CE to sustain prolonged cycling [18]. As shown in Figure 3a, when the average CE is 99.9%, a cell with 20 µm Li can theoretically survive only slightly more than 1000 cycles, whereas a cell with 100 µm Li can reach nearly 5000 cycles. This difference arises because, in ultrathin Li anodes, the limited Li reservoir leaves little room to compensate for irreversible Li loss, making the demand for highly reversible Li plating/stripping much more stringent. Consistent with this analysis, Figure 3b shows that the thick‐Li coin cells commonly used in laboratory studies generally exhibit much better cycling stability than thin‐Li pouch cells under practical conditions [16]. However, this thickness‐dependent trend is not universally valid. Although thicker Li has long been considered advantageous for cycling stability because its larger Li reservoir can better tolerate irreversible Li loss, later studies revealed that this is not always the case. In particular, the choice of electrolyte can fundamentally alter the interfacial evolution by dictating SEI chemistry and Li‐ion transport behavior. With appropriate electrolyte formulations, continuous electrolyte decomposition can be suppressed and a thin, homogeneous, and robust SEI can be established, thereby stabilizing ultrathin Li metal anodes. Liu et al. showed that, in their cell system, reducing the Li thickness from 100 µm (Figure 3c) to 20 µm (Figure 3d) unexpectedly resulted in improved cycling stability [27]. This improvement was attributed to a better balance among Li consumption, electrolyte degradation, and SEI accumulation. As further summarized in Figure 3e, thinner Li metal can promote the formation of a thinner and more uniform SEI layer, whereas thicker Li tends to accumulate a progressively thicker and more heterogeneous interphase, which eventually dominates the cell failure process [27]. These observations suggest that Li thickness alone does not determine cycling durability. Rather, the key lies in whether the electrolyte can induce a stable, low‐resistance interphase. Therefore, optimizing SEI characteristics through rational electrolyte design is essential for enabling ultrathin Li metal anodes to achieve stable long‐term cycling. More detailed interfacial design strategies will be discussed in the following sections.

### 3.2 Diminished Li Reservoir and Structural Instability

**FIGURE 4:**
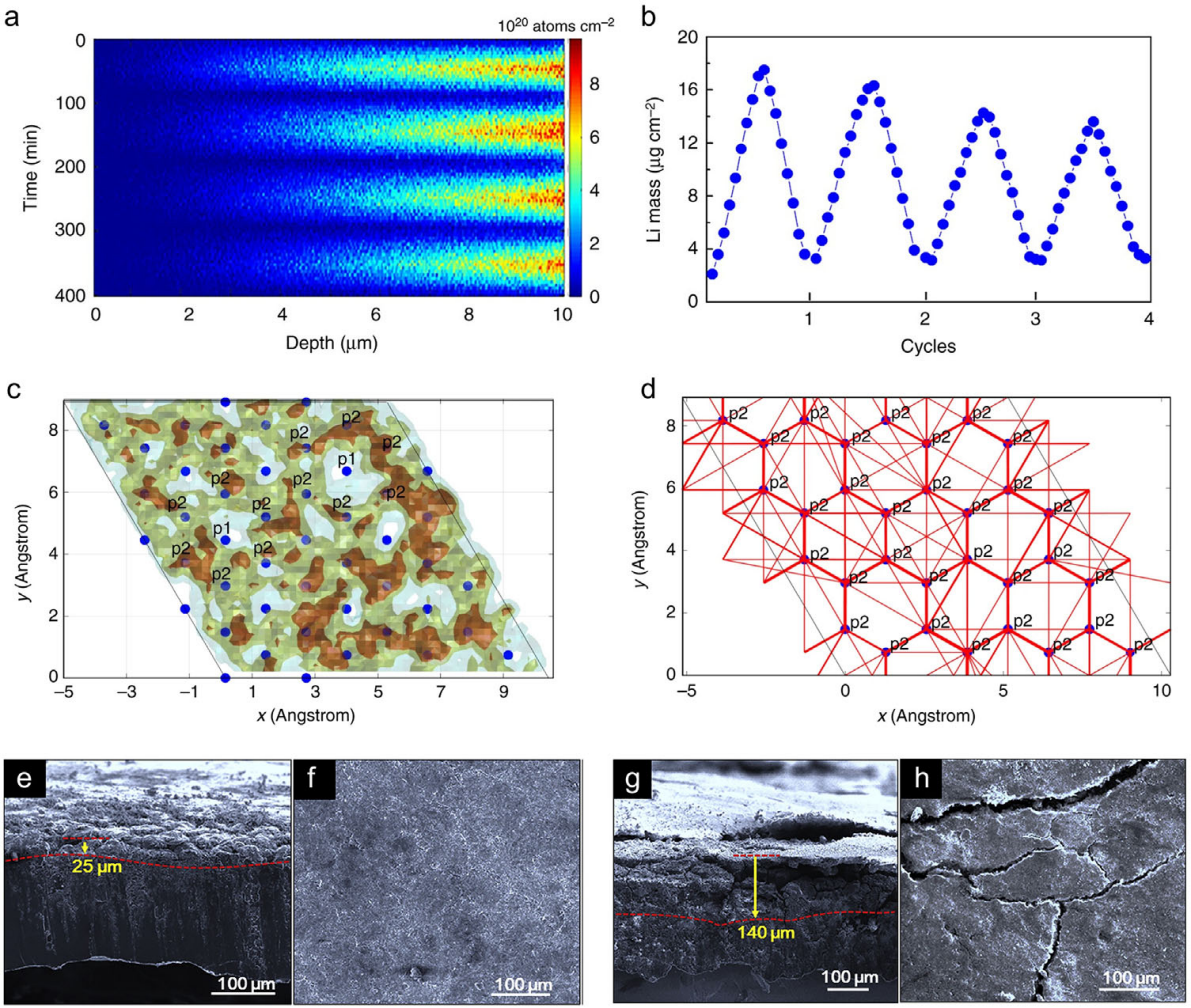
(a) Operando neutron depth profiling during four plating/stripping cycles. (b) Areal Li content in the Cu current collector during the four plating/stripping cycles. (c) Top‐view Li density distributions of a single Li atom diffusing on the Cu (111) surface during a 75 ps simulation at 600 K, showing the stable p2 adsorption sites and the less favorable p1 sites. (d) Diffusion pathways identified from transitions between p2 sites, shown as red lines; line thickness scales with the number of detected transitions. (e) Cross‐sectional morphology of the Li anode and (f) Enlarged view of the Li anode cycled at 1.0 mAh cm^−2^, showing a thin degradation layer and largely preserved bulk Li. (g) Cross‐sectional morphology of the Li anode and (h) Enlarged view of the Li anode cycled at 4.0 mAh cm^−2^, showing a thick degradation layer, severe corrosion, and obvious cracking. (a–d) Reproduced with permission [42]. Copyright 2021, Springer Nature. (e–h) Reproduced with permission [43]. Copyright 2018, Elsevier.

For ultrathin Li metal anodes, the limited Li reservoir makes any form of Li loss far more detrimental than in thick‐Li systems. Beyond dead Li formation and continuous SEI collapse/reconstruction, an additional but often overlooked source of Li consumption arises from the Cu current collector itself. As revealed by operando neutron depth profiling (Figure 4a), Cu is not completely inert during Li plating and stripping, but can take up a considerable amount of Li, part of which becomes irreversibly trapped [42]. In the study by Lv et al., the total Li uptake by Cu reached nearly 20 µg cm^−2^ under certain deposition conditions, and although most of this Li could be reversibly removed during stripping, around 4 µg cm^−2^ remained irreversibly trapped near the Cu interface (Figure 4b) [42]. This directly represents a loss of active Li inventory. Such a process is particularly problematic for ultrathin Li anodes, where the available Li reservoir is extremely limited and even a small amount of irreversible Li loss can lead to a disproportionate reduction in cycle life. The severity of this issue is further amplified by its strong dependence on plating conditions. The amount of Li taken up by Cu increases markedly at aggressive deposition conditions. For thick Li anodes, this hidden Li sink may remain partially masked by the excess Li inventory. In contrast, for ultrathin Li, such parasitic Li consumption will accelerate the fast depletion of the already limited Li source. Mechanistically, operando XRD reveals that Li uptake by Cu does not involve significant dissolution into bulk Cu crystallites, but is instead associated with grain‐boundary‐related regions. This is attributed to the sluggish diffusion of Li in bulk Cu, which necessitates alternative transport pathways. In this context, the rapid surface diffusion of Li (Figure 4c,d) facilitates its migration toward and along grain boundary regions, which act as the primary pathways for Li transport. Together with the DFT results showing that bulk Li insertion into Cu is thermodynamically unfavorable, whereas surface adsorption in the grain boundaries is favorable. Their work inspires efforts into the rational design of Li resistance current collectors.

Another major challenge for ultrathin Li metal anodes is their pronounced susceptibility to mechanical instability and structural degradation during cycling. As areal capacity utilization increases, repeated Li plating/stripping induces large volume fluctuations, which generate substantial mechanical stress, fracture, and delamination within the inherently fragile degradation layer [43, 44, 45]. This trend is clearly reflected by the morphological evolution of cycled Li (200 µm) under increasing areal capacity loading. At a low areal loading of 1.0 mAh cm^−2^, the cycled Li anode largely preserves its bulk structure and only develops a thin degradation layer of about 25 µm, with no obvious cracks observed on the surface (Figure 4e,f). In contrast, when the areal capacity loading is increased to 4.0 mAh cm^−2^, the degradation layer becomes much thicker and more severe corrosion is observed, accompanied by wide and clearly visible cracks (Figure 4g,h) [43]. More importantly, the cracked degradation layer becomes prone to detaching from the underlying bulk Li. These results indicate that higher Li utilization not only intensifies interfacial degradation, but also introduces substantial mechanical instability into the cycled Li anode. For ultrathin Li, such damage is expected to be far more detrimental, because the limited Li thickness provides little tolerance for crack propagation, interfacial delamination, and structural collapse. Once cracking occurs, detached or fractured Li regions can lose electronic contact with the current collector, thereby becoming electrochemically inactive. Meanwhile, the newly exposed Li surfaces continuously react with the electrolyte, further accelerating parasitic side reactions and depleting active Li. Additionally, the formation of fragile and poorly ionically conductive SEI components, such as LiF and Li_2_CO_3_ (10^−10^ to 10^−9^ S cm^−1^ at 100°C) [46], can further aggravate interfacial ion transport resistance and thereby electrochemical performance decay. Overall, the interplay of interfacial degradation, mechanically induced Li isolation, and impedance buildup from resistive SEI formation underscores the necessity of targeted interfacial design strategies. Rational interfacial engineering through electrolyte additives or the direct introduction of artificial interphases is therefore essential for enabling practical ultrathin Li metal anodes.

### 3.3 Challenges of Incorporating Ultrathin Li Metal in ASSLMBs

Building upon the challenges previously outlined, the utilization of ultrathin Li metal anodes, while promising significant potential for increased energy density in Li metal batteries, faces formidable obstacles due to high reactivity, mechanical fragility, and stringent interface requirements. ASSLMBs offer significant advantages in addressing some of these challenges associated with Li metal anodes [47, 48]. The high mechanical strength of SSEs suppresses dendrite growth, enhancing safety by preventing short circuits [49, 50, 51]. Solid electrolytes are generally stable over a wide temperature range. In contrast, liquid electrolytes suffer from limited thermal stability due to the low boiling points and flash points of organic solvents, particularly at elevated temperatures (>80°C) [52, 53]. Additionaly, SSEs also typically exhibit high Li^+^ transference numbers, which help reduce concentration polarization and promote more uniform Li deposition [54, 55, 56, 57].

**FIGURE 5:**
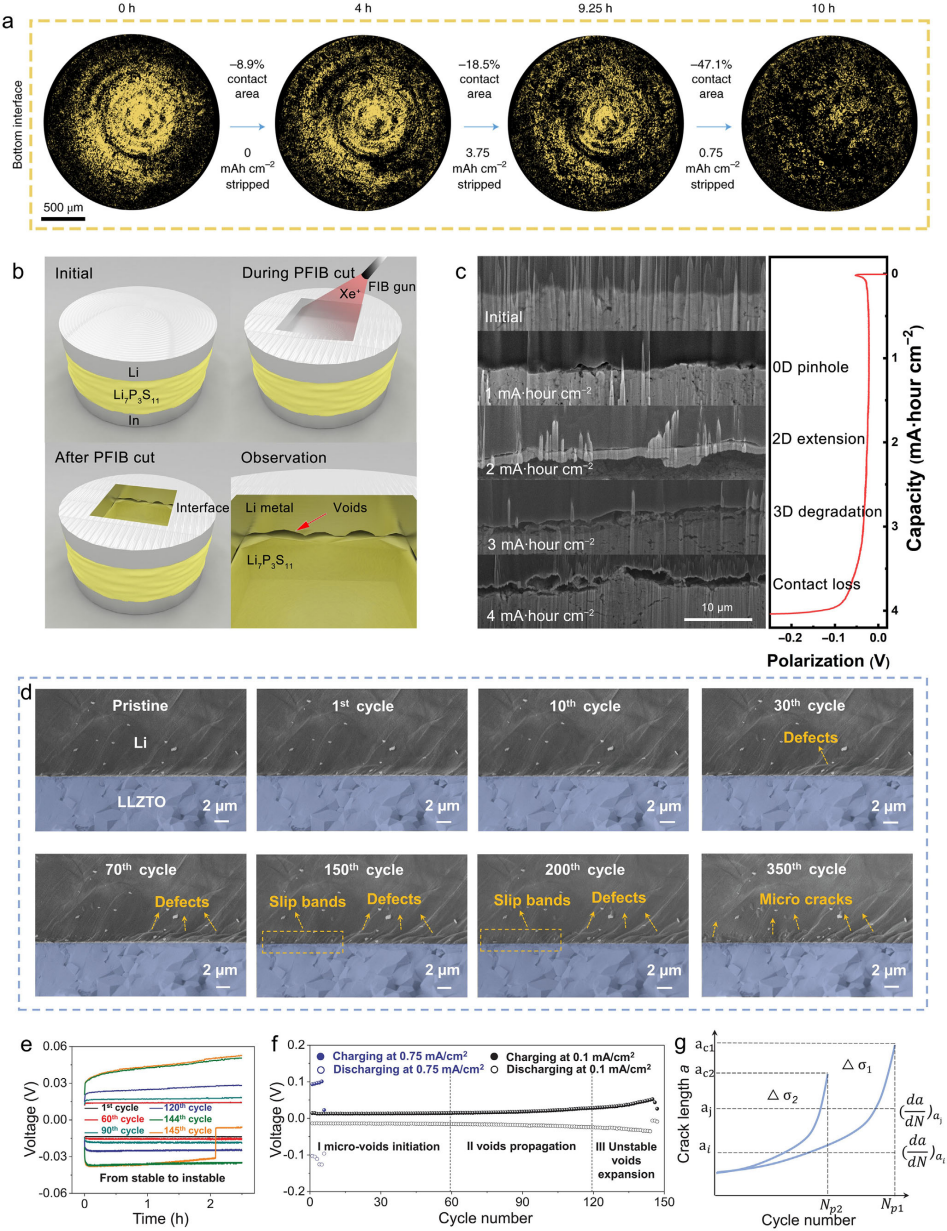
(a) Contact map of the bottom Li|Li_10_SnP_2_S_12_ interface during stripping, showing progressive contact loss and fragmentation associated with void formation. (b) Schematic illustration of the ex situ FIB‐SEM method used to probe interfacial evolution after Li stripping. (c) Polarization curve of the Li‐Li_7_P_3_S_11_‐In half cell during continuous Li stripping at 1 mA cm^−2^, reflecting interfacial Li depletion. (d) Operando SEM images of the Li metal anode during cycling, showing progressive interfacial degradation. (d–g) Reproduced with permission. (e) Charge–discharge profiles of a Li|LLZTO|Li symmetric cell cycled at 0.1 mA cm^−2^ with a capacity of 0.25 mAh cm^−2^. (f) Voltage evolution of symmetric cells cycled at 0.1 and 0.75 mA cm^−2^, and a sudden voltage drop indicates a short circuit. (g) Schematic fatigue crack propagation curve of a metallic material. (a) Reproduced with permission [58]. Copyright 2021, Springer Nature. (b, c) Reproduced with permission [60]. Copyright 2022, AAAS. (d–g) Reproduced with permission [61]. Copyright 2025, AAAS.

However, despite these advantages, in ASSLMBs, the interfacial failure mechanism differs fundamentally from that in liquid‐electrolyte systems because the rigid solid–solid interface cannot dynamically reconfigure to maintain interfacial contact during stripping. This issue has been directly visualized by operando X‐ray tomography, where Figure 5a shows that the initially continuous Li|Li_10_SnP_2_S_12_ contact progressively fragments into small, isolated regions during stripping [58]. Quantitative analysis of the interfacial contact evolution further showed that stripping‐induced contact loss leads to small, isolated contact regions, which greatly enhance current constriction and ultimately drive cell failure.

This mechanistic picture was further clarified for Li|LLZTO‐type interfaces, where discontinuous interfacial contact was proposed to generate voids that induce severe current constriction and strong local current amplification at the remaining contact regions [59]. As a result, void growth was identified as an early precursor to dendrite formation, and its onset was found to be closely correlated with the critical current density for dendrite‐induced shorting. Consistent with this view, ex situ PFIB‐SEM analysis of the buried Li|Li_7_P_3_S_11_ interface further revealed the progressive evolution of stripping‐induced voids [60]. As illustrated by the observation strategy in Figure 5b and the corresponding interfacial morphology in Figure 5c, the initially intimate Li|SSE contact first develops small 0D defects, which then extend laterally into 2D interfacial defects, followed by severe 3D degradation with interconnected cracks, and finally evolve into near‐complete contact loss [60]. This morphological evolution is accompanied by a synchronous increase in polarization, indicating that continued void growth progressively disrupts interfacial transport and accelerates cell failure.

More recently, the interfacial degradation in ASSLMBs has been interpreted from a mechanical fatigue perspective. Operando SEM observations in Figure 5d show that the Li|LLZTO interface progressively evolves from initial defects to slip bands and eventually to microcracks during repeated cycling [61]. The corresponding voltage evolution in Figure 5e,f marks a transition from stable cycling to unstable interfacial behavior. To rationalize this progression, Figure 5g introduces the classical fatigue crack propagation framework of metallic materials, in which both crack length and crack growth rate increase with cycle number until final fracture occurs. By drawing this analogy, the study proposes that interfacial failure in solid‐state Li metal batteries can likewise be divided into three stages: microvoid initiation, void propagation, and ultimately unstable void expansion [61]. In this sense, void formation is not merely a transport‐limited consequence of incomplete Li replenishment, but also a mechanically accumulated fatigue process under cyclic current.

**FIGURE 6:**
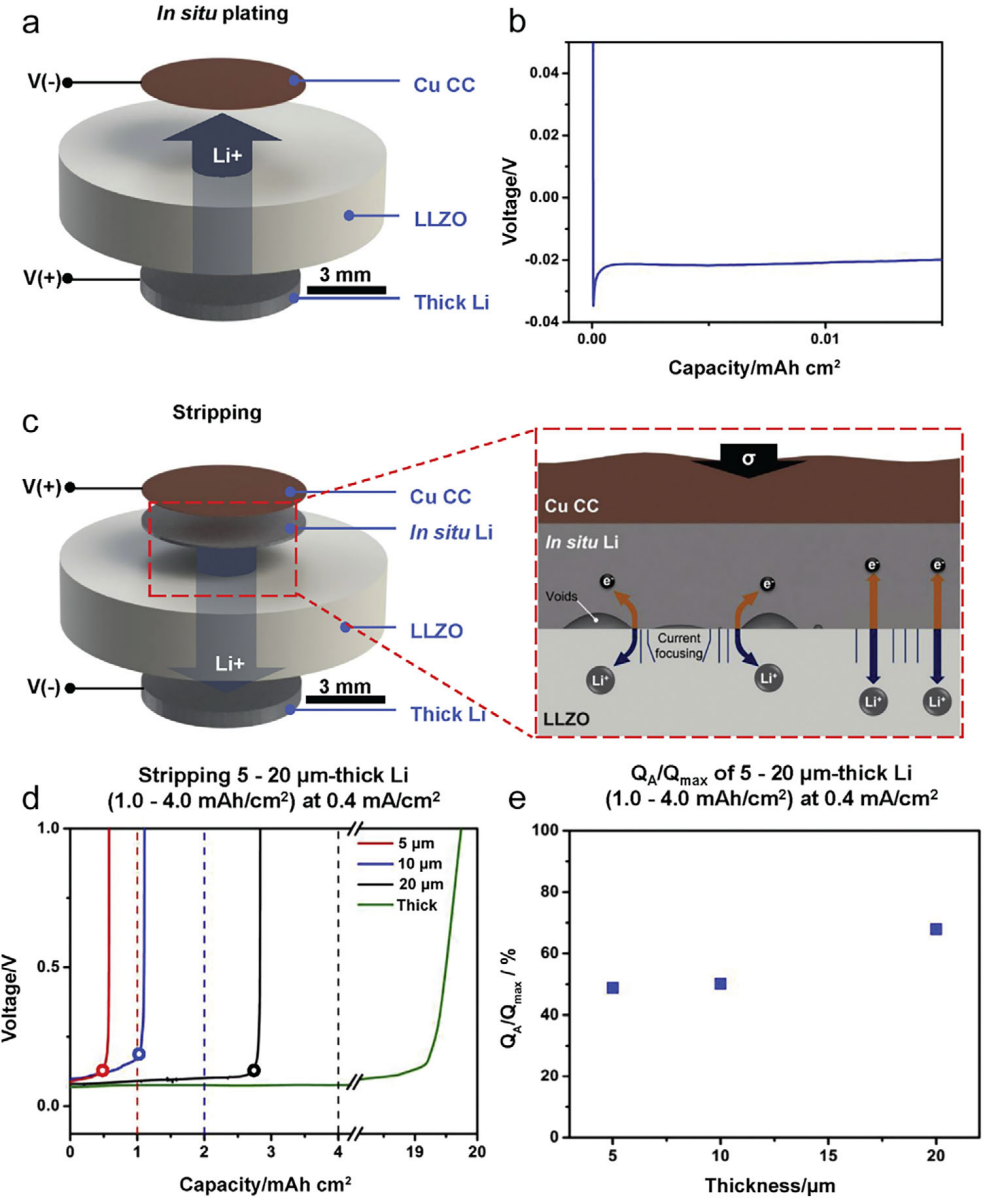
(a) A schematic illustration of a custom cell. (b) voltage profile of in situ plating of Li at 0.05 mA cm^−2^. (c) A schematic illustration of stripping in situ plated Li, including a zoomed‐in Li|LLZO interface. (d) Voltage profiles of 5‐, 10‐, and 20‐µm‐thick in situ Li and 0.75‐mm‐thick Li foil stripped at 0.4 mA cm^−2^, with plated capacities shown as dashed lines in corresponding colors. (e) Q_A_‐to‐Q_max_ ratios for each thickness, as represented in (d). (a–e) Reproduced with permission [62]. Copyright 2022, Elsevier.

The severity of these interfacial challenges might be further amplified when the Li anode is ultrathin. The pronounced vulnerability of ultrathin Li to these interfacial failure modes is further evidenced by a Li‐free half‐cell study using LLZO as the solid electrolyte. In this design, Sakamoto et al. employed a custom cell (Figure 6a,c), where 1.0–4.0 mAh cm^−2^ (5–20 µm) of Li was in situ electrodeposited between a Cu foil current collector (as shown in Figure 6b, indicating the nucleation of Li on the Cu) and LLZO, to analyze the relationship between accessible Li capacity, current density, and Li thickness [62]. As illustrated in Figure 6d, with a thickness of 20 µm, the Li anode retained approximately 70% of its capacity during stripping, whereas 5 and 10 µm Li anodes retained only around 50%. This suggests that the ratio of accessible capacity (Q_A_) to the plated capacity (Q_max_) decreased as the thickness of the Li electrode decreased (Figure 6e) [62]. These results indicate that, as the Li layer becomes thinner, stripping‐induced contact loss becomes substantially more detrimental because a much larger fraction of the limited Li inventory is rendered electrochemically inaccessible. This problem is further aggravated by the reduced ability of thin Li to flow and redistribute along the rigid LLZO interface during stripping, which makes interfacial contact increasingly difficult to maintain [63, 64, 65]. As a result, void formation in ultrathin Li not only disrupts local contact, but also intensifies current constriction, accelerates void expansion, increases interfacial polarization, and ultimately causes a much faster loss of usable capacity than in thicker Li electrodes.

### 3.4 Advanced Strategies to Address the Challenges

In response to these challenges above, strategies focusing on the regeneration of inactive lithium have emerged as promising avenues to improve the practical viability of ultrathin lithium metal anodes. In particular, dynamic self‐repair mechanisms that enable the recovery of electronically isolated lithium (i‐Li) are gaining increasing attention. This section provides a comprehensive overview of recent advancements in these strategies, which aim to address the limitations of ultrathin Li metal anodes and extend their practical application.

**FIGURE 7:**
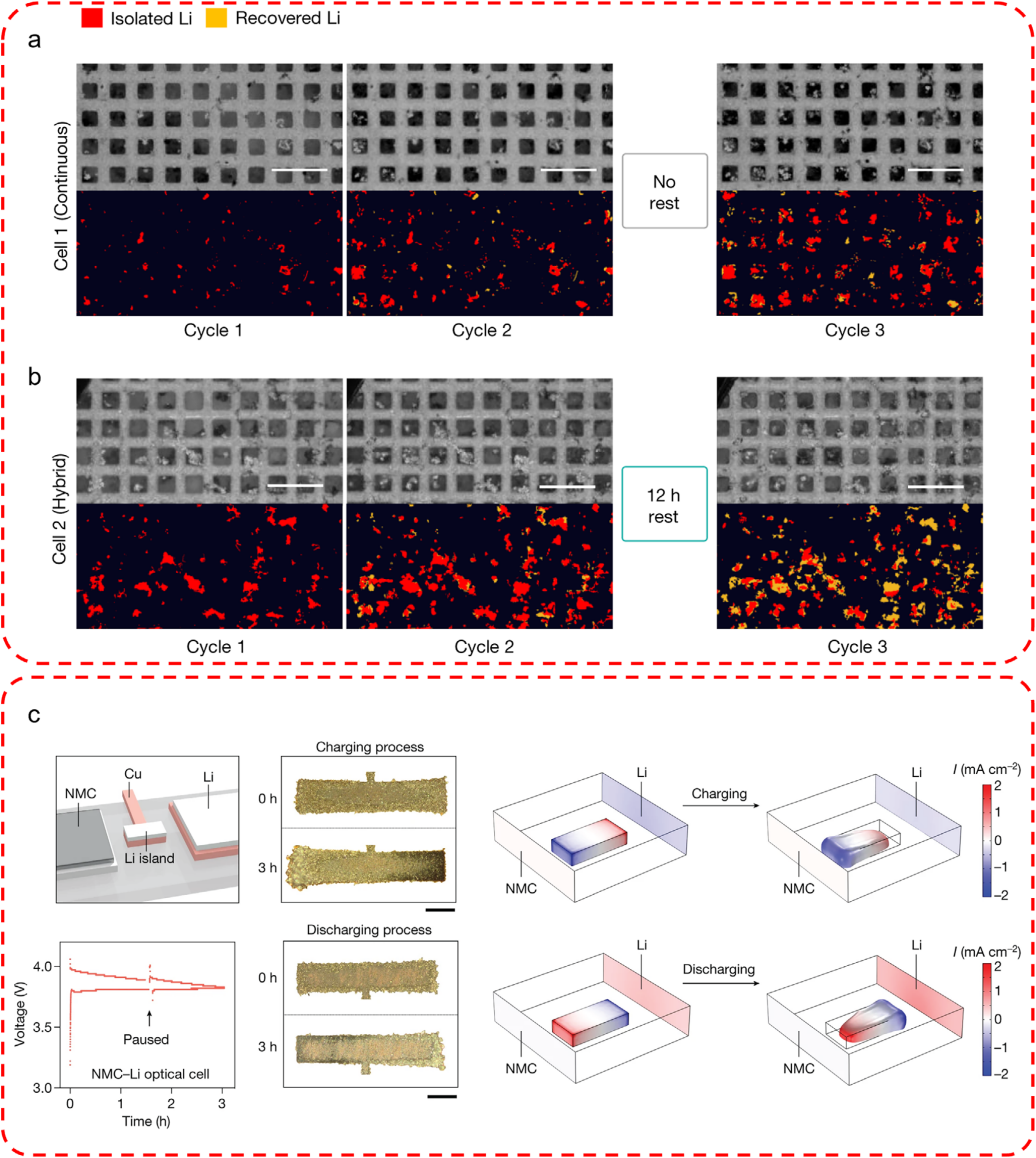
(a) Cell 1, continuous cycling protocol: optical images (top) of Cu mesh in the discharged state for each respective cycle and colormap (bottom) showing the area of isolated Li (i‐Li, red) and recovered Li (r‐Li, yellow) at the end of the first, second, and third cycles. (b) Cell 2, hybrid rest cycling protocol with 12‐h discharged rest between the second and third cycles: optical images (top) of Cu mesh in the discharged state for each respective cycle and colormap (bottom) showing the area of i‐Li (red) and r‐Li (yellow) at the end of the first, second, and third cycles. (c) Morphological evolution of an isolated lithium (i‐Li) island during charge and discharge, including optical cell configuration, voltage response, time‐resolved optical images, and simulated current density distributions, revealing the dynamic migration and electrochemical reconnection of i‐Li under coupled stripping–plating processes. (a, b) Reproduced with permission [66]. Copyright 2024, Springer Nature. (c) Reproduced with permission [67]. Copyright 2021, Springer Nature.

A promising strategy to extend the lifespan of ultrathin Li metal anodes involves the recovery of electronically isolated lithium (i‐Li) through static aging in the discharged state. During this process, cells are rested after full discharge, allowing partial dissolution of the residual SEI (r‐SEI) and re‐exposure of i‐Li. The exposed i‐Li can reconnect with the bulk electrode, thereby increasing the amount of active Li available for cycling and improving CE. Figure 7b,c illustrate this process: at cycle 3, a large fraction of i‐Li was converted into recovered Li (r‐Li, yellow), evidencing effective regeneration. The CE exceeding 100% and titration gas chromatography measurements further confirm that static discharged‐state aging enables substantial i‐Li reactivation. Moreover, operando optical imaging under realistic stack pressure directly visualized this recovery, revealing an R/I > 1 ratio after resting. This behavior originates from the dissolution of the organic components in r‐SEI during rest, which reduces interfacial resistance and facilitates Li reconnection. Overall, discharged‐state static aging provides a simple yet powerful electrochemical protocol to rejuvenate inactive Li, achieving near‐complete capacity recovery and greatly enhancing the long‐term reversibility of Li‐metal batteries [66]. A complementary strategy to extend the lifespan of lithium metal anodes involves the dynamic recovery of electronically isolated lithium (i‐Li) during cycling. In this approach, inactive lithium is not irreversibly lost but can progressively migrate and reconnect with the bulk electrode under an applied electric field through coupled stripping–plating processes [67]. The extent of this reactivation is governed by the geometric characteristics of i‐Li (e.g., length and orientation) and the applied current density, which together determine the rate of lithium reintegration. This mechanism enables the continuous reconnection of isolated lithium and effectively suppresses the accumulation of inactive lithium during cycling. As a result, the electrochemical behavior deviates from conventional systems: in typical Cu–Li cells, Coulombic efficiencies remain below 100% (e.g., ∼94.1%), indicating persistent lithium loss. In contrast, systems exhibiting dynamic lithium recovery can deliver apparent Coulombic efficiencies exceeding 100%, providing direct electrochemical evidence for the reactivation of previously isolated lithium. Overall, these findings demonstrate that dead lithium can function as a dynamically recoverable lithium reservoir rather than being permanently inactive, offering an additional pathway to improve the reversibility of ultrathin Li metal anodes.

## 4 Innovative Technique for Ultrathin Li Metal Production

For practical Li metal batteries, an ultrathin Li metal with an areal capacity of less than 4 mAh cm^−2^ (preferably less than 3 mAh cm^−2^) is required to match the reversible capacity of commonly used Li transition metal oxide cathodes [16, 68], hereby corresponding to a Li metal thickness of below 20 µm (preferably below 15 µm) [25]. The recent advancements in fabricating ultrathin Li foils underscore an increasing focus area, emphasizing the necessity of paying more heed to ultrathin Li metal for practical LMBs design. Based on this understanding, various fabrication strategies can be broadly classified into two types: chemical and physical methods. This section summarizes detailed strategies for preparing ultrathin Li metal from these two perspectives and offers a comparison of their fabricated ultrathin Li metal as a candidate in LMBs.

### 4.1 Chemical Methods

**FIGURE 8:**
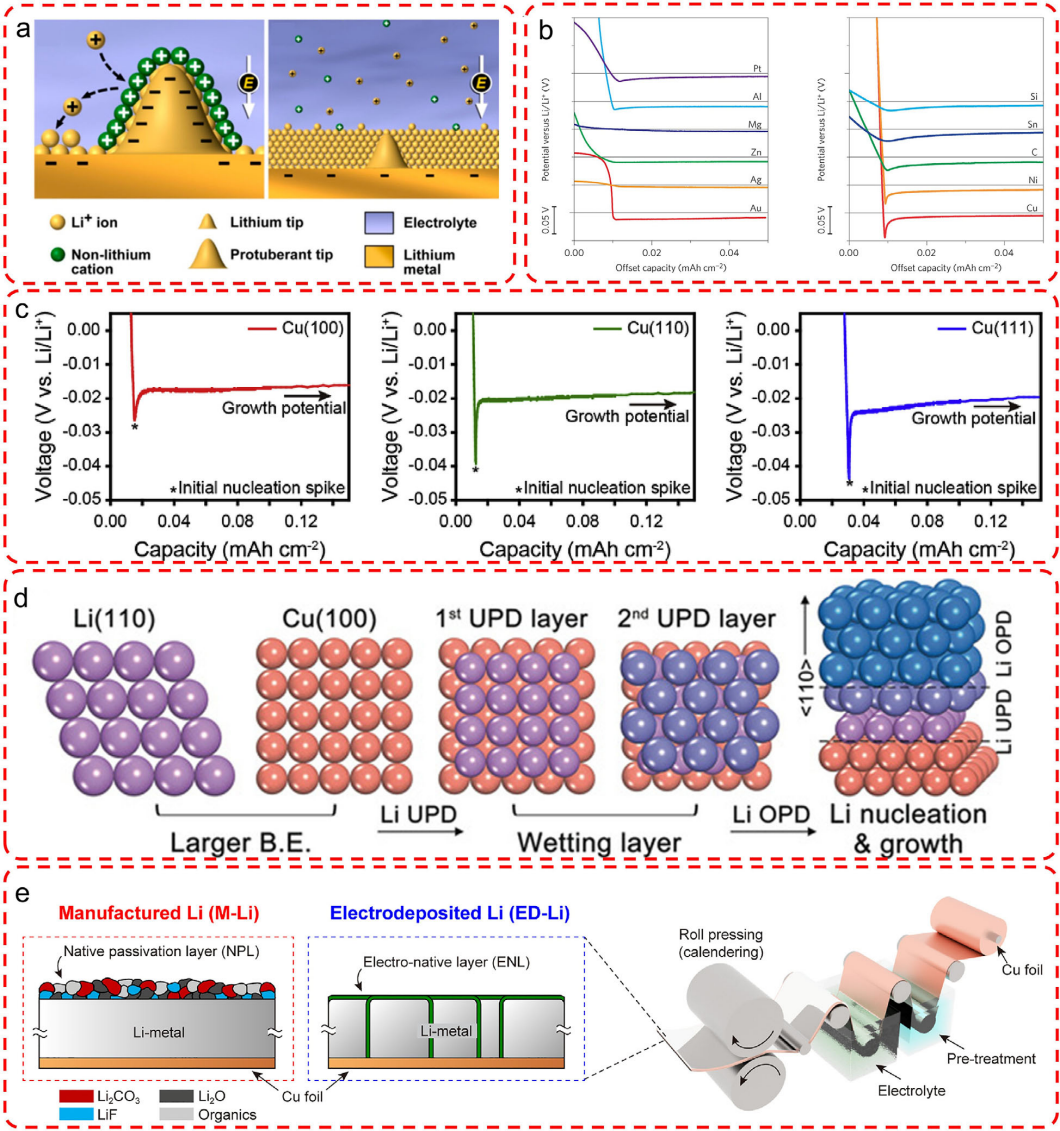
(a) Illustration of the Li deposition process based on the Self‐Healing Electrostatic Shield Mechanism. (b) Voltage profiles of various materials with some solubility (left) and materials with negligible solubility (right) in Li during Li deposition at a current density of 10 µA cm^−2^. (c) Li nucleation on Cu single crystals: Voltage profiles and schematics for Cu(100), Cu(110), and Cu(111) using 1 M LiTFSI DOL/DME (1:1 v/v) with 0.2 M LiNO_3_. (d) Schematics of the structures of Li UPD layers and the crystallographic orientation of deposited bulk Li. (e) The structural distinction between M‐Li and ED‐Li is illustrated with a schematic representation of the electroplating process for large‐area ultrathin Li metal anode laminates. (a) Reproduced with permission [73]. Copyright 2013, American Chemical Society. (b) Reproduced with permission [75]. Copyright 2016, Springer Nature. (c) Reproduced with permission [76]. Copyright 2019, Elsevier. (d) Reproduced with permission [77]. Copyright 2019, Wiley‐VCH. (e) Reproduced with permission [78]. Copyright 2023, Elsevier.

Electrochemical deposition plays an indispensable role in surface engineering technologies, typically employed for the generation of protective, decorative, and/or functional coatings. The fundamental steps of electroplating involve the reduction of metal cations followed by deposition onto a substrate. One of the key advantages of electroplating is the ability to control the thickness of the ultrathin Li layer (0.1 to 20 µm) on the current collector by regulating the deposition time and current density [69, 70, 71]. However, during the electroplating process, the ultrathin Li layer is prone to issues of roughness and high porosity, frequently coupled with filamentous or dendritic growth [72]. To achieve uniform and dense Li plating, electrolyte engineering with specific additives has proven highly effective. These additives regulate Li nucleation and deposition kinetics, thereby suppressing uncontrolled dendrite formation. For instance, incorporating cesium ions (Cs^+^), which possess an equilibrium potential lower than that of Li^+^, can induce an electrostatic shielding effect (Figure 8a) [69, 73]. This effect drives Li^+^ ions to deposit preferentially within concave regions, yielding a flatter and more compact morphology. Alternatively, alkali metal ions such as sodium (Na^+^), with a deposition potential close to that of Li^+^, can form a Na‐Li solid‐solution phase that alters the Li growth pathway and effectively mitigates dendritic propagation [74]. Collectively, these additive‐mediated electrochemical routes provide a controllable and scalable approach to fabricate smooth, dense, and ultrathin Li metal layers suitable for practical Li metal batteries.

The choice of substrate plays a pivotal role in achieving a smooth deposition of ultrathin Li. The uneven Li electro‐deposition on heterogeneous substrates has been a longstanding issue in Li metal anodes [75, 79]. From the standpoint of intrinsic solubility between the substrate material and Li metal, a substrate that exhibits appreciable solubility with Li (Figure 8b), such as gold (Au), silver (Ag), zinc (Zn), and magnesium (Mg), should be preferred for Li deposition [75]. This is because a solid‐solution interfacial layer can form prior to Li metal deposition, which lowers the nucleation barrier and facilitates subsequent Li growth by providing energetically favorable nucleation sites. Conversely, materials with no solubility in Li metal (Figure 8b), like Cu, nickel (Ni), C, tin (Sn), and Si, exhibit noticeable overpotentials that are detrimental to Li deposition [75]. It is worth noting that the crystallographic orientation relationship between the substrate and Li is another key factor governing the initial Li nucleation behavior, enabling preferential nucleation on specific crystallographic orientations through surface structure modulation. For instance, the crystallographic orientation of Cu substrates significantly influences lithium nucleation and growth behavior. Studies reveal that the Cu(100) plane demonstrates the lowest nucleation overpotential and the highest growth potential (Figure 8c), indicating its superior ability to facilitate uniform Li deposition compared to Cu(110) and Cu(111). This behavior arises from the Cu(100) plane's lower energy barrier for Li nucleation and its favorable surface characteristics for stable Li growth [76]. Furthermore, the Cu(100) surface can promote Li growth through favorable crystallographic orientation relationships (e.g., Kurdjumov–Sachs or Nishiyama–Wasserman‐type relationships) with Li(110). This facilitates the formation of a Li underpotential deposition (UPD) layer that guides bulk Li growth along the (110) direction (Figure 8d). This alignment reduces the nucleation barrier and ensures uniform, dendrite‐free Li deposition, highlighting the potential of Cu(100) surfaces to enhance the performance and stability of Li metal anodes [77].

Recently, with advancements in electrolyte design, it has been discovered that a surface‐derived SEI, also referred to as an electrolyte‐derived native layer (ENL), forms on ultrathin Li layers electrodeposited in localized high‐concentration electrolytes (LHCEs). These electrolytes are typically formulated by diluting highly concentrated Li‐salt solutions with weakly solvating hydrofluoroether solvents [78]. The ENL formed on ultrathin Li layers electrodeposited in this electrolyte promotes fast and homogeneous Li nucleation, leading to smooth and uniform Li plating. This understanding offers valuable guidance for the rational design of electrolytes in Li electrodeposition systems.

Chemical etching—with its high selectivity, low equipment costs, suitability for batch production, and controllable etching rate—has been widely used as a common method to control the depth and verticality of surface etching on metal materials and to prepare thin films [80]. By designing stable 3D structures on the surface of the current collector via chemical etching (for example, in situ etched nanostructured CuO on commercial Cu foil), the dendritic reaction potential can be reduced to guide uniform Li nucleation and deposition [81]. Moreover, chemical etching can also serve independently as an excellent potential candidate method for ultrathin Li production. This is achieved by selecting a solution capable of undergoing a Lewis acid‐base reaction on the surface of Li metal, thereby etching to reduce the thickness of the Li metal. For instance, the spontaneous chemical etching reaction that occurs at the solid‐liquid interface between naphthalene (Naph) and Li in a Naph/DME (1,2‐dimethoxyethane) solution can produce a smooth Li surface with a thickness of less than 15 µm [82]. The controllable reaction rate and excellent adjustability of this method highlight its potential for the continuous, large‐scale production of ultrathin Li.

### 4.2 Physical Methods

**FIGURE 9:**
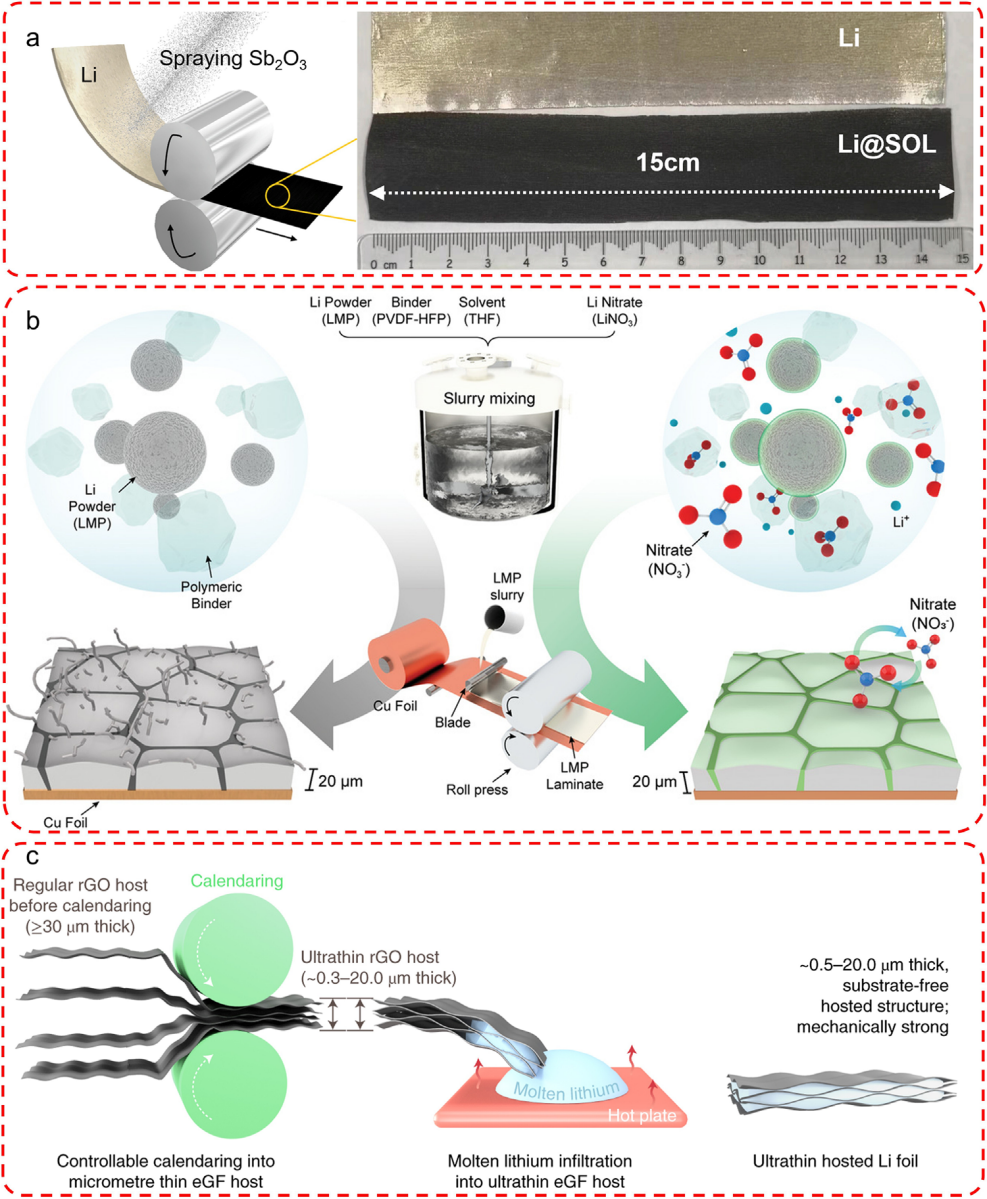
(a) Schematic illustration of in situ nano‐passivation layer formation by spraying Sb_2_O_3_ powder, accompanied by a photo comparison of unprotected Li metal vs. a nano‐composite layer (abbreviated as SOL) Sb_2_O_3_‐protected Li. (b) Schematic of the LMP‐based composite electrode fabrication process, encompassing slurry mixing with intentional LiNO_3_ addition for LN‐LMP composites, solution casting, and roll‐press lamination. (c) Controllable calendaring compresses eGF films into thin hosts, followed by molten Li infiltration and annealing to produce thin, strong, and thickness‐tunable Li@eGF foils. (a) Reproduced with permission [44]. Copyright 2020, Elsevier. (b) Reproduced with permission [88]. Copyright 2021, Wiley‐VCH. (c) Reproduced with permission [25]. Copyright 2021, Springer Nature.

Mechanical rolling of Li foil is one of the most facile and scalable methods for the production of ultrathin Li. The stickiness and poor mechanical plasticity of Li metal lead to unrestricted dislocation motion during the deformation of the Li foil, posing a significant obstacle to the production of ultrathin Li metal by mechanical rolling [83, 84]. In recent years, with the development of more precise rolling equipment and control systems, many industrial mechanical rolling processes can now produce ultrathin Li less than 20 µm [85]. However, the use of ultrathin Li metal prepared by these methods is still limited in laboratory‐level academic research because these pure ultrathin Li metals are prone to holes and cracks caused by the uneven electrochemical stripping of Li metal in the battery, leading to severe morphological evolution and the division of the original ultrathin Li metal into multiple “dead” Li parts [25]. Recent work provides a method of pre‐spraying the Li surface with Sb_2_O_3_ before mechanical rolling (Figure 9a) [44]. The self‐compacting separator (4.5 µm) formed by the reaction on the Li surface helps to achieve stable deposition on the rolled ultrathin Li (15.5 µm). The Sb_2_O_3_ powder can also reduce the stickiness between the rolling machine and the Li, making the rolled ultrathin Li easy to separate from the rolling machine and prevent Li rupture. Spraying Sb_2_O_3_ onto the Li surface triggers interfacial redox reactions, in which Sb_2_O_3_ is reduced by Li to generate Sb and Li_2_O, followed by alloying between Li and Sb to produce Li_3_Sb. These sequential reactions give rise to an in situ formed nanocomposite interphase, denoted as a nano‐composite layer (SOL). The resulting SOL layer possesses a partially consolidated structure that remains permeable to the liquid electrolyte.

In addition to simply reducing the thickness by mechanically rolling Li foil, ultrathin Li metal prepared by mechanically rolling spherical Li metal powder (LMP) slurry with a larger surface area can effectively control the thickness (<20 µm) and reduce local current density during the deposition process [86]. However, uncontrolled morphological evolution of the LMP surface remains a major challenge during repeated Li plating/stripping [86, 87]. The incorporation of additives into the LMP slurry to modify the LMP surface can mitigate the issue of morphological evolution. For example, the 20 µm ultrathin Li metal prepared from an LMP slurry containing LiNO_3_ enriches the LMP surface with Li_3_N and LiN_x_O_y_ compounds, which can prevent the loss of Li on the part of LMP and maintain their spherical shape (Figure 9b) [88].

Indeed, an alternative strategy involves fabricating a host framework by mechanical rolling, followed by molten Li infusion via an edge‐contacting method. In this approach, Li is accommodated within the internal channels of the rGO framework, while the host maintains a micrometer‐scale thickness. The resulting Li foils exhibit tunable thicknesses in the range of 0.5–20 µm (Figure 9c) [25]. This host‐assisted strategy provides a practical route for the scalable production of ultrathin Li metal anodes.

**FIGURE 10:**
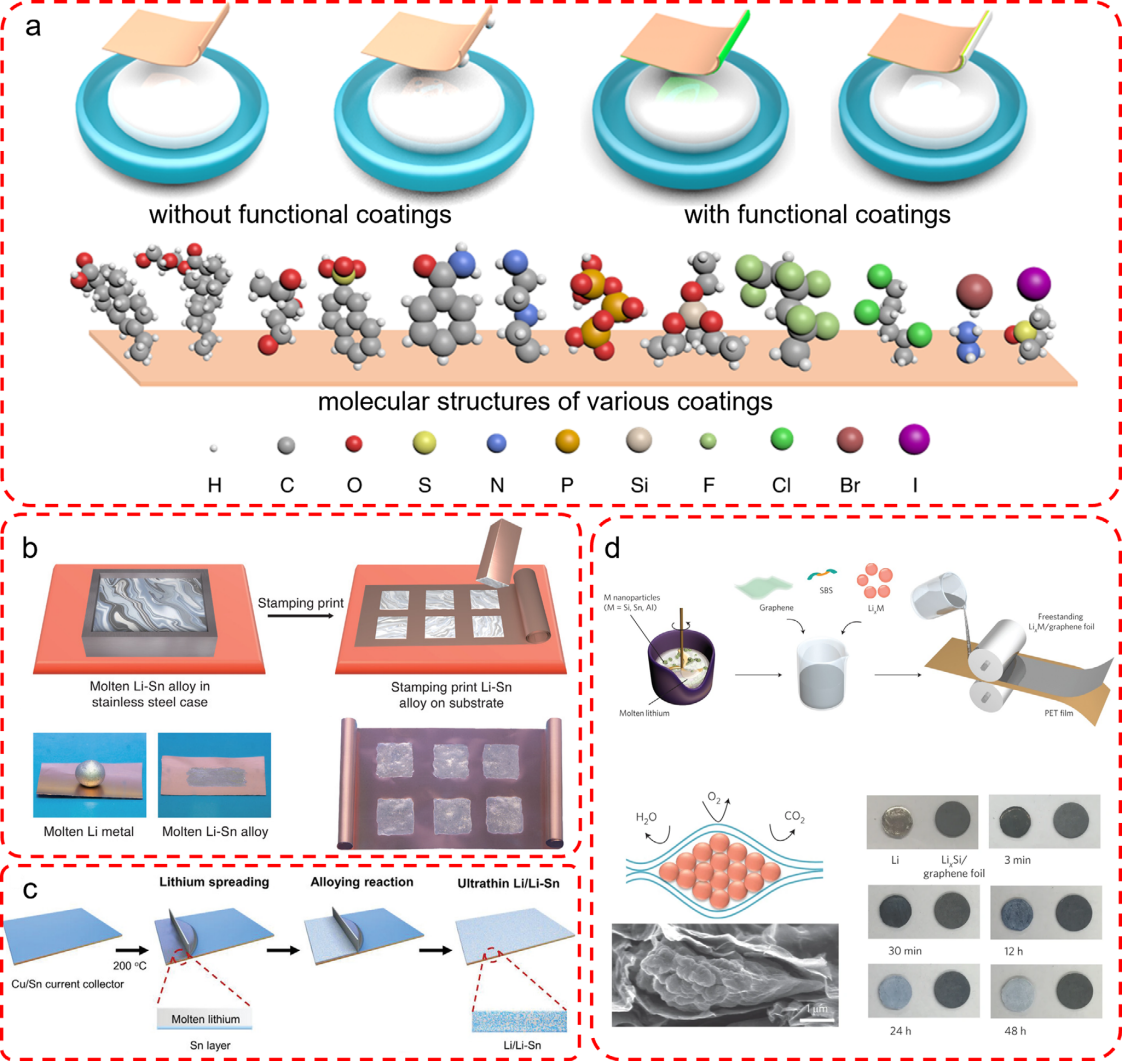
(a) Schematic of ultrathin Li layer formation on lithiophobic substrates, showing molten Li contact with a Cu substrate without and with functional coatings, alongside the molecular structures of various coatings. (b) Schematic of the stamping process, showing Li–Sn alloy stamping with a Cu‐based postmark and inkpad, wettability comparison of molten Li and Li–Sn alloy on Cu foil, and printed Li–Sn alloy patterns on Cu foil. (c) Schematic of a doctor‐blade casting fabrication process of ultrathin Li||Li–Sn electrode. (d) Schematic illustration of nanoparticle synthesis, slurry casting, and calendaring, along with graphene encapsulation of LixM clusters. SEM image confirms the confined structure, and air‐exposure tests demonstrate the enhanced environmental stability of the composite foil compared to Li metal. (a) Reproduced with permission [95]. Copyright 2019, Springer Nature. (b) Reproduced with permission [99]. Copyright 2021, Wiley‐VCH. (c) Reproduced with permission [101]. Copyright 2021, Wiley‐VCH. (d) Reproduced with permission [103]. Copyright 2017, Springer Nature.

Compared to mechanical rolling, which employs relatively simple, room‐temperature equipment (e.g., a roll press), coating molten Li directly onto a current collector involves greater complexity, higher costs, and safety concerns due to the need for elevated temperatures (>350°C) and additional heating of both Li and current collector substrates [89, 90]. However, despite these challenges, molten Li coating offers unique advantages, such as flexibility in adjusting the Li metal layer thickness and improved interfacial engineering. By enhancing wettability through strategies [91, 92, 93], such as coating current collectors with organic functional groups (such as ─COOH, ─SO_3_H, ─NH_2_, ─NH) or incorporating reactive elements (such as In, Mg, S, Se, etc.), it becomes feasible to uniformly deposit ultrathin Li metal layers onto various lithiophobic substrates (Figure 10a) [94, 95, 96]. Moreover, molten Li coating methods enable alloy formation (e.g., Li–Sn, Li–Zn), significantly improving adhesion between Li and the substrate, thereby creating mechanically robust and chemically stable interfaces (Figure 10b,c) [97, 98, 99, 100, 101]. Additionally, innovative techniques such as magnetic‐field‐assisted molten Li deposition allow precise control and fabrication of ultrathin Li metal layers down to 10 µm (Figure 10d) [102]. These distinctive benefits illustrate the potential of molten Li coating as a versatile approach for the practical fabrication of advanced ultrathin Li metal anodes, despite its inherent operational complexities.

One of the challenges associated with the direct coating method of ultrathin Li metal layer fabrication is the relatively weak adhesion between metallic Li and the current collector, leading to easy delamination of the Li metal layer. This issue can be effectively mitigated by introducing alloying strategies using metals such as Sn, Zn, Ag, and Al, which can react with Li to form lithiophilic interfacial phases. The alloying process can enhance the wettability of molten Li on the surface of these metals, thereby creating a tightly coupled structure between Li metal and the current collector. For instance, a Cu‐Sn alloy or a Sn layer on a Cu current collector can separately achieve a uniform and controllable Li layer by stamping a Li‐Sn alloy or molten Li scraping onto the Cu foil substrate, respectively (Figure 10b,c). In addition to interfacial alloying, studies from Cui's group have demonstrated that integrating alloy components within a conductive carbon framework can further enhance structural stability [103]. For example, Li‐containing alloy nanoparticles (e.g., Li–Si, Li–Sn, or Li–Al) encapsulated within graphene matrices can form freestanding composite foils with improved interfacial stability and mechanical integrity (Figure 10d). The graphene layers act as a protective barrier, effectively suppressing side reactions with ambient air and enabling the material to remain stable under ambient conditions.

Recently, Pasta et al. presented a comprehensive and innovative techno‐economic analysis method that integrates detailed battery performance targets with industrial‐scale production considerations, specifically tailored to evaluate ultrathin Li metal anodes for solid‐state batteries. Distinct from traditional approaches, this method quantitatively defines the maximum permissible Li thickness (17 µm or less) necessary to reach volumetric energy density targets exceeding 1000 Wh L^−1^ while meeting stringent CE criteria of over 99.9% [15]. The study rigorously compares multiple Li fabrication techniques (including extrusion, liquid‐based methods, electrodeposition, and vapor‐phase processes) and identifies thermal evaporation (TE) as the most viable for scalable manufacturing. Notably, the analysis incorporates detailed kinetic modeling of TE deposition processes, considering practical deposition rates above 100 nm s^−1^, optimization of roll‐to‐roll substrate widths (expanded from conventional 1.2 m up to 3.0 m to reduce capital expenditures), and achieving Li utilization efficiencies surpassing 90%. Moreover, the techno‐economic model includes extensive sensitivity analyses examining the global impacts of lithium carbonate price fluctuations, regional electricity cost variations, carbon‐emission offsets, and labor cost disparities. This multifaceted approach yields an intricate breakdown of cost structures, strategically highlighting pathways for significant cost reductions and advancing the practical feasibility of ultrathin Li metal anodes in large‐scale gigafactory production [15].

## 5 Challenges of Ultrathin Li Metal|Electrolyte Interface

### 5.1 Fundamentals of Ultrathin Li Metal|Electrolyte Interface

Due to the chemically aggressive nature of Li metal, the majority of electrolytes are thermodynamically unstable with Li metal, inducing the formation of an SEI layer on the Li metal surface. A stable SEI layer can passivate and protect Li metal from further corrosion reactions. However, the natively formed SEI layer is typically fragile, which leads to repeated cracking/reforming of the SEI layer due to large volume change during the Li plating/stripping process. The collapse of the SEI layer exposes fresh Li metal to further reaction with the electrolyte, ultimately resulting in the fast depletion of Li metal and electrolyte [104]. Additionally, the heterogeneity of the SEI layer will provide Li metal nucleation sites, which can trigger the growth of lithium dendrites. In turn, the growth of Li dendrite can also collapse the SEI layer, accelerating the depletion of the Li metal and electrolyte, as well as the formation of a thick, resistive SEI layer. It is a formidable challenge to enable ultrathin Li metal anode stable and durable operation due to the serious Li dendrite growth and severe Li metal|electrolyte continuous loss. Thus, to develop highly efficient ultrathin Li metal anode and corresponding high‐energy‐density batteries, it is essential to maintain durable Li metal|electrolyte interface stability and mitigate Li dendrite growth. The ultrathin Li metal|electrolyte interfacial challenges will be discussed separately from the perspectives of liquid electrolytes (LE) and SSE. The corresponding strengthening strategies are also summarized and discussed.

### 5.2 The Ultrathin Li Metal|LE Interface

**FIGURE 11:**
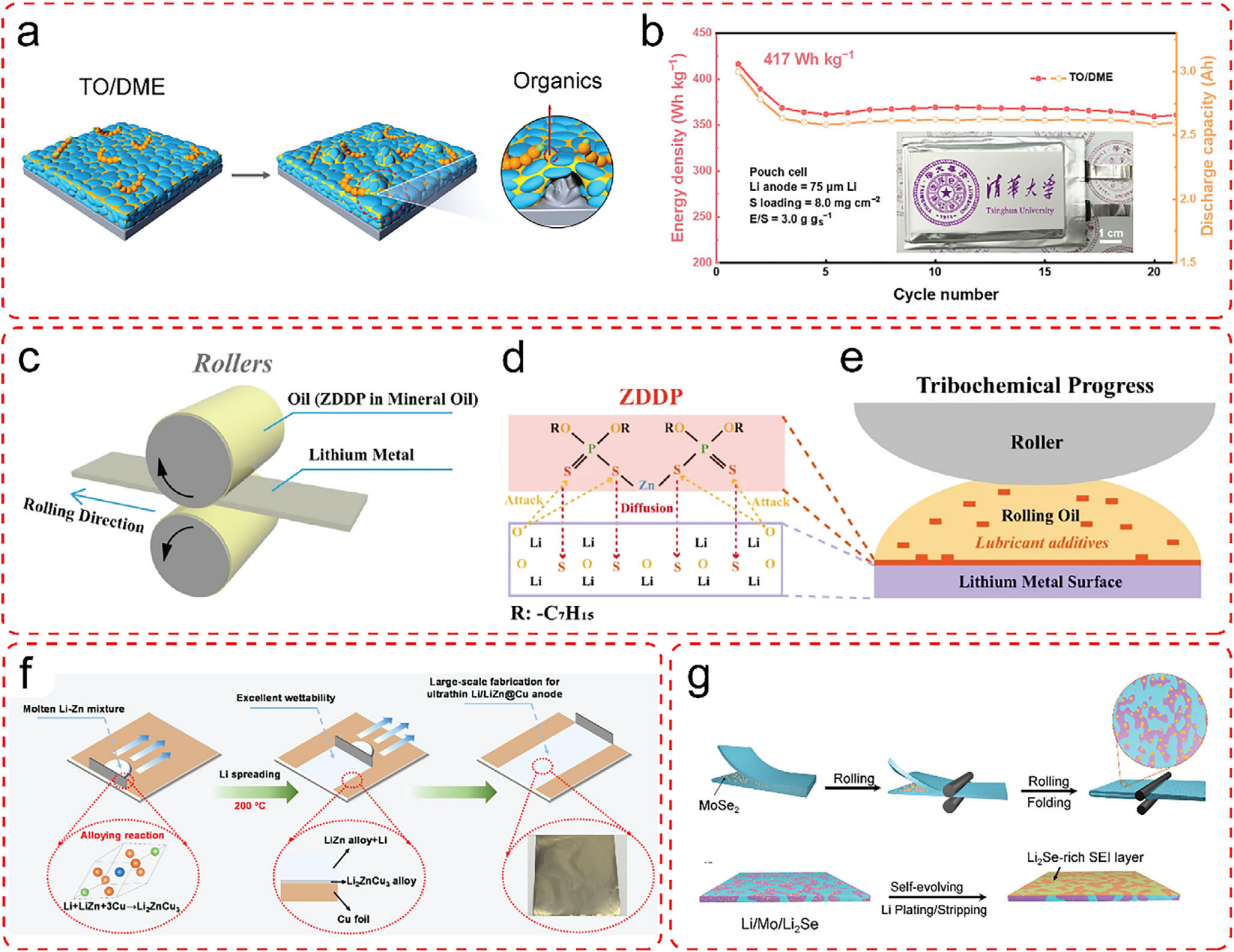
(a) Scheme illustrating the evolution of SEI with TO/DME electrolyte and (b) the cycling performance of a Li‐S pouch cell with TO/DME electrolyte. (c) Schematic diagram of the preparation of Li@ZDDP using ZDDP additives. (d) Schematic of the mechanochemical reaction under the catalysis of friction and alkali metal ions. (e) Schematic diagram of the reaction of ZDDP molecules with Li metal during the rolling process. The schematic illustrations of (f) preparation of Li/LiZn@Cu and (g) ultrathin Li/Mo/Li_2_Se. (a, b) Reproduced with permission [106]. Copyright 2023, Wiley‐VCH. (c–e) Reproduced with permission [108]. Copyright 2023, Springer Nature. (f) Reproduced with permission [109]. Copyright 2024, Springer Nature. (g) Reproduced with permission [117]. Copyright 2024, Wiley‐VCH.

Exploiting ultrathin Li metal is the ultimate avenue toward high‐energy‐density LMBs. However, the notorious Li dendrite growth and serious interfacial side reactions of Li metal with LE severely fetter the practical usage of ultrathin Li metal‐based LMBs. Currently, interfacial modulation is the most widely recognized effective strategy to stabilize ultrathin Li metal, which is mainly achieved via electrolyte additives [105, 106] and artificial SEI construction [107, 108, 109]. Lu's group [105] reported a dual‐additive electrolyte refers to 1 M LiPF_6_ in fluoroethylene carbonate/ethyl methyl carbonate (FEC/EMC, 3:7 v/v) containing 1 wt.% tris(pentafluorophenyl)borane (TPFPB) and 3 wt.% LiNO_3_ for practical Li metal battery construction. A robust outer Li_2_O‐rich SEI was formed on the Li metal surface during cycling, benefiting from the decomposition of NO_3_^−^ in the solvation structure. This SEI layer can regulate the uniform Li deposition and suppress the side reactions between the electrolyte and Li metal. To investigate the practical usage prospect of the dual‐additive electrolyte, the 4.0 mAh cm^−2^ Li||NCM811 cell based on ∼46 µm thin Li metal was assembled and tested. Using the control electrolyte of FEC/EMC, the cell can only cycle for less than 30 cycles. In sharp comparison, the cell with a dual‐additive electrolyte demonstrated significantly improved cycling stability over 100 cycles, even with a high cut‐off voltage of 4.5 V and a lean electrolyte (∼3.4 g Ah^−1^). To further improve the mechanical stability of the SEI layer, 1,3,5‐trioxane (TO) was proposed to optimize the ether electrolyte by Zhang and co‐workers [106]. TO molecules possess a high polymerization capability, which can facilitate an organic‐rich SEI layer formation with enhanced mechanical stability (3.7 GPa), compared to the SEI derived from DOL/DME (1.9 GPa). As a result, the consumption of Li metal, polysulfide as well as electrolyte is suppressed owing to the organic‐rich SEI layer (Figure 11a). The Ah‐level Li‐S pouch cell with Li foil anode (75 µm) and high‐loading cathode (8.0 mg cm^−2^) were assembled to evaluate the TO/DME electrolyte. The Li‐S pouch cell exhibits a high energy density of 417 Wh kg^−1^ and can cycle over 20 cycles (Figure 11b). The interplay among electrolyte additives, Al current collectors, bulk electrolyte chemistry, and interfacial stability has recently attracted increasing attention. Understanding the cross‐talk between cathode, current collector, and ultrathin Li anode is particularly important for enabling wide‐temperature operation of Li metal batteries. For instance, boron‐containing additives can coordinate with PF_6_^−^ and suppress electrolyte decomposition, thereby improving the cycling performance of Ni‐rich cathodes at room and low temperatures [110]. Liu et al. further proposed a cross‐coupled electrolyte design, in which solvent‐derived radical intermediates diffuse to the cathode to suppress DFOB^−^ oxidation and gas evolution, while salt‐derived radical intermediates migrate to the anode to trigger interfacial polymerization, forming a polymeric SEI [111]. This process leads to the formation of an ∼8 nm thick amorphous, single‐layer SEI enriched with B–F‐containing polymer species on the Li surface, while simultaneously mitigating gas evolution at the cathode. The resulting SEI exhibits low Li^+^ transport activation energy, enabling a 2D planar Li deposition behavior. As a result, the anode‐free cell demonstrates stable cycling for 100 cycles at 100% depth of discharge (DoD) and 250 cycles at 80% DoD, with a capacity retention of ∼80%. However, many boron‐containing additives exhibit pronounced degradation at elevated temperatures. Xie et al. quantitatively analyzed the Al content deposited on Li metal using ICP‐OES and revealed that boron‐containing electrolytes significantly accelerate Al current collector corrosion, especially at high temperature, as evidenced by the highest Al deposition on the Li surface [112]. To address this issue, tris(2,2,2‐trifluoroethyl) borate (TFEB) was identified as an effective additive to suppress Al corrosion. Meanwhile, TFEB reduces cathode–electrolyte interfacial reactions and promotes the formation of an electronically insulating cathode–electrolyte interphase (CEI), enabling improved cycling stability over a wide temperature range. Beyond additive engineering, molecular‐level solvent design provides new opportunities to enhance the interfacial stability of ultrathin Li metal. In conventional electrolytes, Li^+^–solvent interactions are dominated by ion–dipole coordination with O‐ or N‐based ligands, which facilitates salt dissolution but often hinders interfacial charge transfer. To balance salt solubility and ion–dipole interactions, Wu et al. designed monofluorinated alkane solvents, in which the Lewis basicity of F‐containing ligands enables high salt solubility (> 2 mol L^−1^). The introduction of weak F–Li^+^ coordination in the primary solvation sheath facilitates Li plating/stripping kinetics and enables ultrathin Li metal (∼20 µm) to achieve energy densities exceeding 700 Wh kg^−1^ [113]. To promote the formation of inorganic‐rich SEIs on Li metal, researchers have continuously developed fluorinated electrolytes, weakly solvating electrolytes, (localized) high‐concentration electrolytes (HCEs/LHCEs), and additive‐enhanced electrolyte systems.To further rationalize electrolyte design, Fan and co‐workers proposed a unified descriptor based on cation/anion–solvent affinity (α_s_ and β_s_). In this framework, weaker cation–solvent affinity (more negative α_s_) promotes ion‐pair dissociation, while stronger anion–solvent affinity (more positive β_s_) favors the formation of anion‐dominated solvation structures. Solvents with balanced affinity toward both Li^+^ and anions thus enable optimized solvation structures, leading to highly efficient Li plating/stripping behavior. Guided by this principle, approximately 150 candidate solvents were screened, identifying electrolyte systems with Coulombic efficiencies exceeding 99.5% [114]. Alternatively, Ji et al. introduced a microemulsion electrolyte design that departs from conventional Li^+^ solvation regulation. In this system, liquid–liquid interfacial tension between microemulsion droplets and carbonate solvents drives the directional migration of fluorinated domains toward both electrodes, forming fluorine‐rich interphases simultaneously. The accumulation of fluorinated species in the form of micellar structures (IM‐F@AM‐F) results in LiF‐rich SEI formation and uniform Li deposition, delivering an average Coulombic efficiency of ∼99.2% in Li||Cu cells [115]. As discussed above, ultrathin Li anodes are particularly vulnerable under ultrafast charging (UFC) conditions, where repeated volume fluctuations can fracture the SEI, leading to dendrite formation and rapid Li consumption. To address this challenge, Xu and co‐workers designed an MTP solvent featuring a six‐membered bidentate structure with planar‐aligned lone‐pair electrons [116]. This planar‐aligned electron channel (PAEC) enables strong orbital overlap between ligand orbitals and Li^+^ empty orbitals, facilitating electron transfer to Li^+^ while maintaining strong solvation. As a result, interfacial electron transport becomes continuous rather than localized, promoting uniform Li deposition and suppressing dendrite formation. The MTP‐based electrolyte enables 4C ultrafast charging (full charge within 15 min) and stable cycling over 100 cycles, highlighting a new strategy to enhance interfacial charge‐transfer kinetics for ultrathin Li metal anodes.

On the other hand, directly constructing an artificial interface layer on the Li metal surface is an effective approach to stabilize the ultrathin Li metal and LE interface. In 2022, Sun and co‐workers [107] proposed the fabrication of high‐performance ultrathin Li metal via the electrodeposition method. They designed a cross‐linked polymer‐coated Cu foil substrate to support ultrathin Li metal. The rigid and flexible artificial polymer layer greatly improved the CE of ultrathin Li metal to 98.7% over 550 cycles at 0.2 mA cm^−2^. Consequently, the 10 µm ultrathin Li metal shows prolonged cycling over 1300 h at 3 mA cm^−2^. The Li‐S batteries with ultrathin Li metal also demonstrate enhanced cycling stability over 300 cycles, with a high specific capacity of greater than 600 mAh g^−1^, owing to the minimized side reactions of Li metal with electrolyte and polysulfide. Later, Chen's group [108] reported the fabrication of freestanding ultrathin Li metal strips via the interfacial friction‐induced tribochemical reaction of zinc dialkyldithiophosphate (ZDDP) with Li metal (Figure 11c–e). A thin (∼450 nm) organic/inorganic interface layer with high hardness (0.84 GPa) and Young's modulus (25.90 GPa) can be generated on a Li metal surface. This greatly facilitates the scalable production of ultrathin Li metal as thin as 5 µm. Meanwhile, the Li metal|electrolyte interface was also stabilized, thus affording dendrite‐free Li deposition. The symmetric cells with 100 µm Li@ZDDP exhibit a long‐term stable cycling over 1400 cycles at 1.5 mA cm^−2^, 1.5 mAh cm^−2^ and 1700 cycles at 18 mA cm^−2^, 1.5 mAh cm^−2^. The Li||LFP full with 15 µm Li@ZDDP and high‐loading (10 mg cm^−2^) cathode also delivers a long‐term cycling stability over 350 cycles at 5 C with a high capacity retention of >80%. Lu's research group [109] proposed the fabrication of a hierarchical ultrathin Li/LiZn@Cu anode by the doctor‐blading molten Li metal on a Cu substrate aided by the introduction of Zn (Figure 11f). The formed LiZn alloy facilitates homogeneous Li deposition and significantly strengthens the SEI layer, thereby minimizing side reactions between Li/LiZn@Cu and the electrolyte. Additionally, the assembled 0.16 Ah‐level single‐layer Li/LiZn@Cu||LCO pouch cell achieves a superior energy density of 284.0 Wh kg^−1^ even under harsh cycling conditions with low N/P ratio (∼1.35) and lean electrolyte (1.83 g Ah^−1^). It is worth noting that the introduction of the Cu substrate inevitably reduces the overall gravimetric energy density of the Li metal anode. Recently, Xia et al. [117] reported a facile and scalable production of ultrathin Li/Mo/Li_2_Se anode (10‐40 µm) via the MoSe_2_‐promoted mechanical rolling process at room temperature (Figure 11g). The in situ generated Mo and Li_2_Se in the composite anode not only enhances mechanical strength, enabling the scalable production, but also promotes uniform Li electrodeposition. Li_2_Se migrated upward during cycling, forming a Li_2_Se‐rich SEI layer on the composite Li metal surface, due to its ionically conductive and electronically insulative property of Li_2_Se (Figure 11g). The Li_2_Se‐reinforced fast‐ion‐transport SEI layer effectively regulated the uniform Li deposition and minimized the side reactions between Li metal and electrolyte. As a result, the Li/Mo/Li_2_Se demonstrates high‐rate performance, long‐term cycling stability as well as superior anti‐pulverization capability. Furthermore, the single‐layer Li||LCO pouch cell with one double‐sided cathode also demonstrated a high initial areal capacity of 3.15 mAh cm^−2^ at 0.25 C with a high capacity retention of 90.1% over 305 cycles, and even enduring the lean electrolyte of ∼3.92 g Ah^−1^.

### 5.3 The Ultrathin Li Metal|SSE Interface

ASSLMBs have aroused enormous attention due to their high safety and high energy density. Over the past few decades, tremendous efforts have been devoted to developing high‐performance SSEs with high ionic conductivity and wide electrochemical stability windows. However, the relatively poor mechanical/electrochemical/chemical stability at the Li metal|SSEs interface remains a critical challenge that limits cell performance. Greater attention should be focused on mitigating interfacial issues to improve the cycling performance of ASSLMBs. Absolutely different from cells using LE with a solid|liquid interface, Li ion diffusion and its redox reaction with active material and electrons in ASSLMBs occur exclusively at the contacted solid|solid interface. Therefore, it is crucial to maintain consistent interface contact to ensure the stable operation of the charge transfer reaction, which is the prerequisite for constructing high‐performance ASSLMBs. It is worth noting that the substantial volume expansion of Li metal during the Li plating/stripping process can degrade the interfacial contact, inducing the formation of voids or defects. This significantly hampers the Li^+^ transport across the interface and consequently deteriorates the cell cycling performance. From this perspective, developing elastic SSEs for fabricating the electrolyte|electrode interface is much more promising. Solid polymer electrolytes show greater advantages in interfacial contact due to their excellent flexibility. Another significant issue contributing to the considerable interfacial resistance between Li metal and SSE is the notable side reaction between Li metal and SSE. Most SSEs show poor chemical stability with Li metal due to the highly reactive nature of Li metal, inducing serious Li metal|SSE side reactions and formation of a gradual thickening of the resistive SEI layer on the Li metal surface. The electrochemical instability at the Li metal|SSE interface, which results in uneven lithium deposition, is also a critical interfacial issue that needs to be addressed. Thus, ensuring mechanical/chemical/electrochemical stability at ultrathin Li metal|SSE interface is critically vital for truly achieving high‐energy‐density ASSLMBs due to the limited Li metal available. The currently reported strategies for stabilizing the Li metal|SE interface can be mainly categorized into 3D micro‐structural design of Li metal and interface layer modulation.

#### 5.3.1 Three‐dimensional Structural Design of Ultrathin Li Metal

Continuous and effective electrode/electrolyte mechanical contact is crucial for guaranteeing the stable operation of ASSLMBs. Due to the fact that an infinite volume expansion of Li metal during the electrodeposition process, a large mechanical stress will be generated at the Li metal|electrolyte interface, which can result in voids formation even crack the SSE. The hindrance of ion transport in void regions induces Li ions to deposit only at sites where Li metal is in close contact with the electrolyte. The local electric field induces the growth of Li dendrites and their penetration across the SSE [118]. Initial attempts have been devoted to enhancing the Young's modulus of SSEs to withstand the interface stress and suppress the Li dendrite growth. However, recent works show that Li dendrite can still proliferate within rigid LLZO‐based SSEs, despite LLZO's shear modulus reaching 60 GPa, one order of magnitude higher than that of Li metal (1.6 GPa) [119]. Therefore, interfacial stress relaxation is crucial to ensure the stable operation of ASSLMBs. 3D‐architectured Li metal design is currently regarded as one of the most effective strategies to alleviate the stress accumulation. In 2018, Hu's group [120] reported a 3D porous ion‐conductive garnet‐type Li_7_La_2.75_Ca_0.25_Zr_1.75_Nb_0.25_O_12_ (LLCZN) scaffold with a tuned thickness ranging from 20 to 150 µm to host Li metal to ensure the durable Li metal|LLCZN interfacial stability. By tuning the thickness of the 3D LLCZN scaffold, the amount of Li metal can be controlled. They found that the 3D LLCZN scaffold can also effectively regulate uniform Li deposition and mitigate the substantial volume expansion of Li metal during cycling. Later, Li's group [121] proposed Li metal deposition/stripping via Coble creep using a 3D mixed ion/electron conductor (MIEC) architecture with electronic and Li‐ion insulators (ELI) root to stabilize Li metal. With the MIEC/ELI scaffold, Li metal was deposited and stripped via interfacial diffusion creep with fast stress relaxation and minimal interfacial contact with SE. Hence, the electrochemical/mechanical stability of ASSLMBs can be improved greatly, enabling the highly reversible Li plating/stripping with dendrite‐free Li deposition while the ELI can suppress Li metal deposition at the root of MIEC host thus effectively mitigating the Li metal|SE interfacial side reactions. The achieved Li||LFP ASSLMBs exhibit almost no degradation over 50 cycles and the specific gravimetric capacity of the composite Li metal reaches 900 mAh g^−1^. Notably, to truly achieve the high‐energy‐density ASSLMB, the thickness of the 3D scaffold should be precisely controlled to minimize the sacrifice of cell volumetric/gravimetric energy density. Additionally, the preparation process of the 3D host for Li metal should be simplified and made cost‐effective to meet the demands of practical applications.

#### 5.3.2 Ultrathin Li Metal|SSE Interface Layer Modulation

**FIGURE 12:**
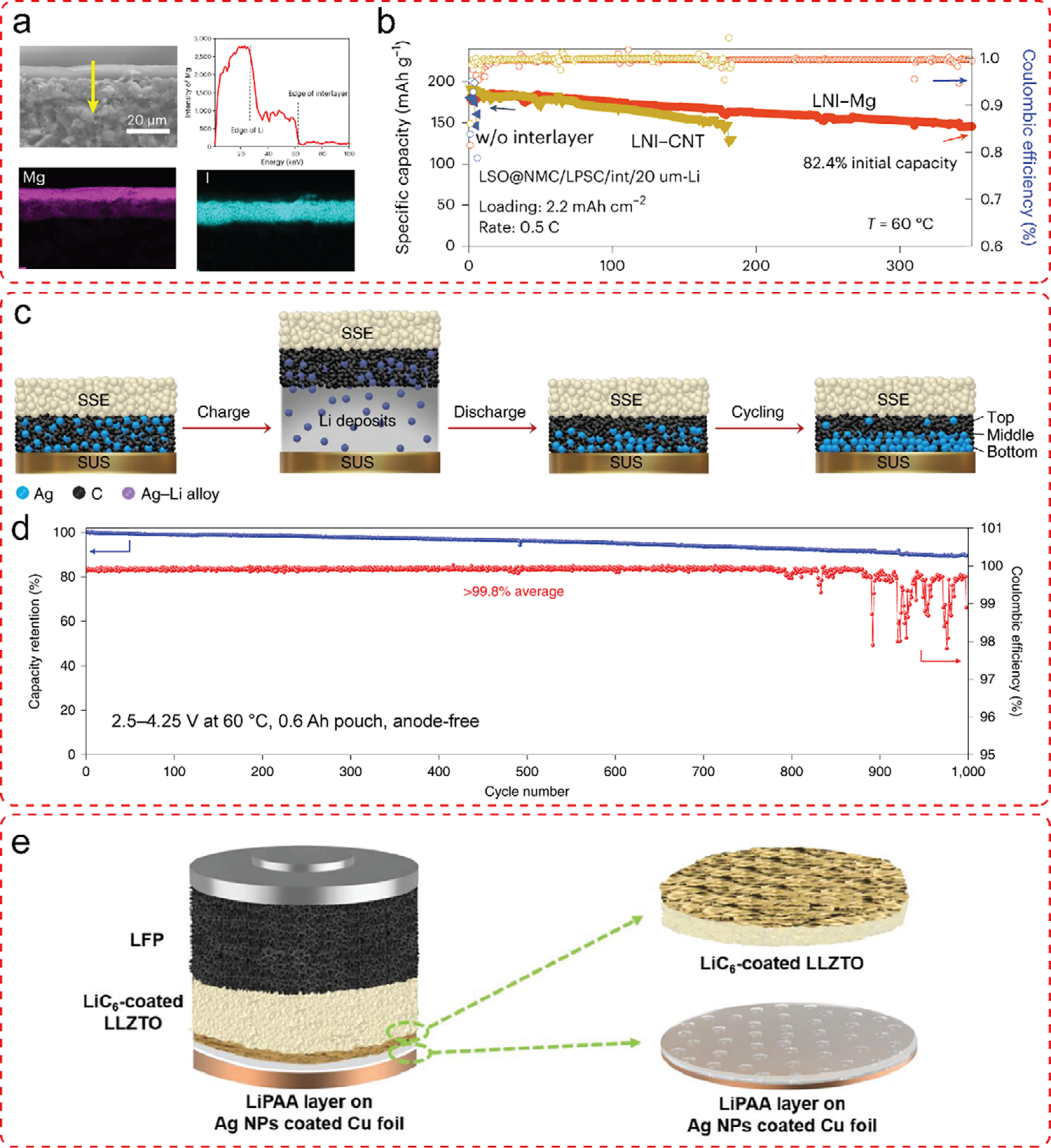
(a) SEM elemental line scan and elemental mapping of activated Li|LNI–Mg interface in Li|LNI–Mg/LPSC|Li cells and (b) the cycling performance of Li||NCM811 ASSLMB (2.2 mAh cm^−2^) with 20 µm ultrathin Li at 0.5 C. (c) Schematic illustration of Li plating/stripping on the current collect with Ag‐C modification layer and (d) the long‐term cycling performance of Ag‐C|LiNi_0.90_Co_0.05_Mn_0.05_O_2_ pouch cell at 0.5 C. (e) Schematic of LLZTO‐based anode‐free cell. (a, b) Reproduced with permission [132]. Copyright 2023, Springer Nature. (c, d) Reproduced with permission [133]. Copyright 2020, Springer Nature. (e) Reproduced with permission [134]. Copyright 2023, Wiley‐VCH.

Voids formation at the Li metal|SSE interface is a key factor degrading the cycling performance of ASSLMBs, which is prone to generate particularly when the Li stripping rate is much larger than its replenishing rate. The voids formation can induce local high current density and thus promote the dendritic Li metal growth [59]. It was reported that the local current density around the voids could be as high as 1000 mA cm^−2^ even if the cell average current density is only 0.5 mA cm^−2^. The significant local current density increase will result in the formation of hotspots, resulting in the reduction of SSE and the notorious Li dendrite growth. Although applying high stack pressure can suppress the voids formation to some extent, it is extremely impractical to develop ASSLMBs relying heavily on external pressure from a long‐term perspective. Incorporation of a modification layer at the Li metal|SSE interface has been regarded one effective strategy to improve the interfacial contact and enhance the Li metal wettability. The modification layer can reduce interfacial resistance via mitigating the voids and facilitating the fast charge transfer at the Li metal|SSE interface even under a low stack pressure. In stabilizing the solid|solid interface via the introduction of an interfacial modification layer, Hu's group are the pioneers. In 2017, they proposed a thin (20 nm) lithiophilic Al interface layer, prepared utilizing the vapor deposition method, to improve the contact of Li metal with LLZO SSE [122]. The Al modification layer significantly decreased the interfacial resistance from 950 to 75 Ω cm^2^ at 20°C. Additionally, Ge [123], Au [124], Ga [125], and Mg [126] were also reported efficient materials for Li metal|LLZO SSE interfacial modification. Despite those modifications, Li dendrite growth still persists, and a short circuit can occur during prolonged cycling or under relatively high current densities. The primary reason of Li dendrite growth currently accepted is the unignorable electronic conductivity of the SSE itself. It has been demonstrated that grain boundaries or defects provide pathways for electronic conduction due to the much higher electronic conductivity in these imperfect areas [127, 128]. Particularly, high applied voltage and elevated temperature can lead to a sharp leap in electronic conductivity, which further raises the risk of electronic leakage across the SSE and promotes Li metal nucleation and growth along the electron conductive pathways. In order to fundamentally block the electron conductive pathway at the interface, Lee et al. [128] designed a LiF/Ag bilayer at the Li metal|LLZO interface with a thickness of ∼300 nm, where the lithiophobic LiF can act as the electron‐blocking layer passivating the electron transport while the lithiophilic Ag layer affords excellent Li metal wettability. With the bilayer modification, the critical current density of Li symmetric cells increased to 3.1 mA cm^−2^ at 60°C. However, the low ionic conductivity of LiF is not conducive to the interfacial Li ion transfer due to its high Li^+^ transfer energy barrier [129]. Later, Sung et al. [130] designed a 3.25 µm nanoporous 3D highly lithiophilic Li_3.75_Si‐CNT MIEC interlayer at the Li|Li_6_PS_5_Cl metal interface. With the robust modification layer, the Li is deposited between the current collector and the interlayer, avoiding the direct contact of Li metal with SSE to some extent. The achieved solid‐state Li||NCM811 with 30 µm ultrathin Li metal manifests a prolonged cycling stability over 200 cycles with a high‐capacity retention of 88.9% at 60°C. It is worth mentioning that their work could not fundamentally mitigate the side reactions of Li metal with SE. Li metal still deposits at the SSE interface, particularly when there is a large Li plating capacity, due to the electronic conductivity of the interface layer, and Li|SSE side reaction. To fundamentally alleviate the interfacial side reactions, a Mg_16_Bi_84_ layer with a thickness of 0.43 mm by the cold press method was rationally constructed at the Li||Li_6_PS_5_Cl interface by Wang and his co‐workers [131]. Triple interlayer composed of LiMgS_x_‐Li_3_Bi‐LiMg was in situ generated during cycling, which enables the homogeneous Li deposition at the LiMg surface and growth into porous Li_3_Bi to alleviate the stack pressure accumulation. Simultaneously, the ion‐conductive and electron‐insulating LiMgS_x_ layer protects the SSE from the Li metal reduction and welds the SSE to the Li_3_Bi interlayer. Both the interfacial side reactions and the Li dendrite growth were effectively suppressed. The critical current density of Li|Li_6_PS_5_Cl|Li increased significantly from 0.4 to 2.6 mA cm^−2^. The Li|Li_6_PS_5_Cl|LiNiO_2_ cell with the Mg_16_Bi_84_ modification layer exhibits a high areal capacity of 11.1 mAh cm^−2^ with a high energy density of 310 Wh kg^−1^, even with a low stack pressure of 2.5 MPa. This work provides an effective strategy paving the way for the commercialization of ASSLMBs. Recently, the same group [132] systematically investigated the interlayer design principle at the Li|SSE interface. They designed a micro‐sized porous lithiophobic Li_7_N_2_I–Mg interlayer with a thickness of 18.5 µm at the Li|LPSCl metal interface. During the activation, gradient electronic conductivity was formed due to the migration of Mg to the Li metal side, which greatly enhanced the Li dendrite suppression capability (Figure 12a). They believed that achieving a balance of lithiophobicity, electronic/ionic conductivity, and porosity of the interlayer is crucial for enabling stable Li plating/stripping at high Li plating capacities. The micro‐sized interlayer enables the Li|LPSCl|Li symmetric cells to cycle for over 100 h at a high current density of 4 mA cm^−2^ with a capacity of 4 mAh cm^−2^. Moreover, the Li||NCM811 cell with 20 µm ultrathin Li also demonstrates a high areal capacity of 2.2 mAh cm^−2^ with a high‐capacity retention of 82.4% over 350 cycles at 0.5 C and 60°C, even with a low stack pressure of ∼1 MPa (Figure 12b). This work is a landmark of development of ASSLMBs up to date, which eliminates the heavy reliance on high external stack pressure.

Anode‐free ASSLMBs with cathode as the sole source of Li is a highly attractive and advanced technology for high‐energy‐density and safe energy storage due to eliminating free Li. In 2020, Lee et al. [133] reported a 5 µm thick Ag‐C nanocomposite layer on the stainless steel current collector for anode‐free ASSLMBs, where the Ag layer can regulate the uniform Li deposition and while the carbon layer can protect LPSCl away from Li metal (Figure 12c). The Li plating/stripping reversibility was thus greatly improved. The 0.6 Ah‐level Ag‐C||LiNi_0.90_Co_0.05_Mn_0.05_O_2_ pouch cell demonstrates a high energy density of 900 Wh L^−1^ and an ultralong lifespan stable cycling over 1000 cycles with a superior capacity retention of 89% and a high average Coulombic efficiency (>99.8%) at 0.5 C and 60°C (Figure 12d). Recently, Huang's group [134] reported a flexible lithiated polyacrylic acid (LiPAA) polymer layer (∼1.2 µm) modified Cu foil with pre‐planted Ag nanoparticles for Li_6.5_La_3_Za_1.5_Ta_0.5_O_12_ (LLZTO)‐based anode‐free ASSLMBs (Figure 12e). The LiPAA polymer layer can keep the intimate contact with LLZTO and accommodate the large interfacial stress variation during the cycling process. Meanwhile, the Li‐Ag alloy can regulate the uniform and dense Li deposition. The assembled LiPAA‐Ag/Cu||LFP ASSLMBs demonstrated a high CE of 99.7% after stable operation for 120 cycles with a superior capacity retention of 90.3% at 0.1 C.

The first strategy offers a distinct advantage by addressing the interfacial contact challenges between the Li anode and the SSE during ultrathin Li metal fabrication. Compared with pure Li metal, Li‐based alloys exhibit lower diffusion barriers. This characteristic, combined with a reduced deposition flux, facilitates Li diffusion along the electrode surface and effectively suppresses dendrite formation [135]. Ultrathin Li alloy anodes can be prepared by introducing alloying elements into molten Li metal, as exemplified by the Li‐Sn alloy coating technique mentioned earlier.

**FIGURE 13:**
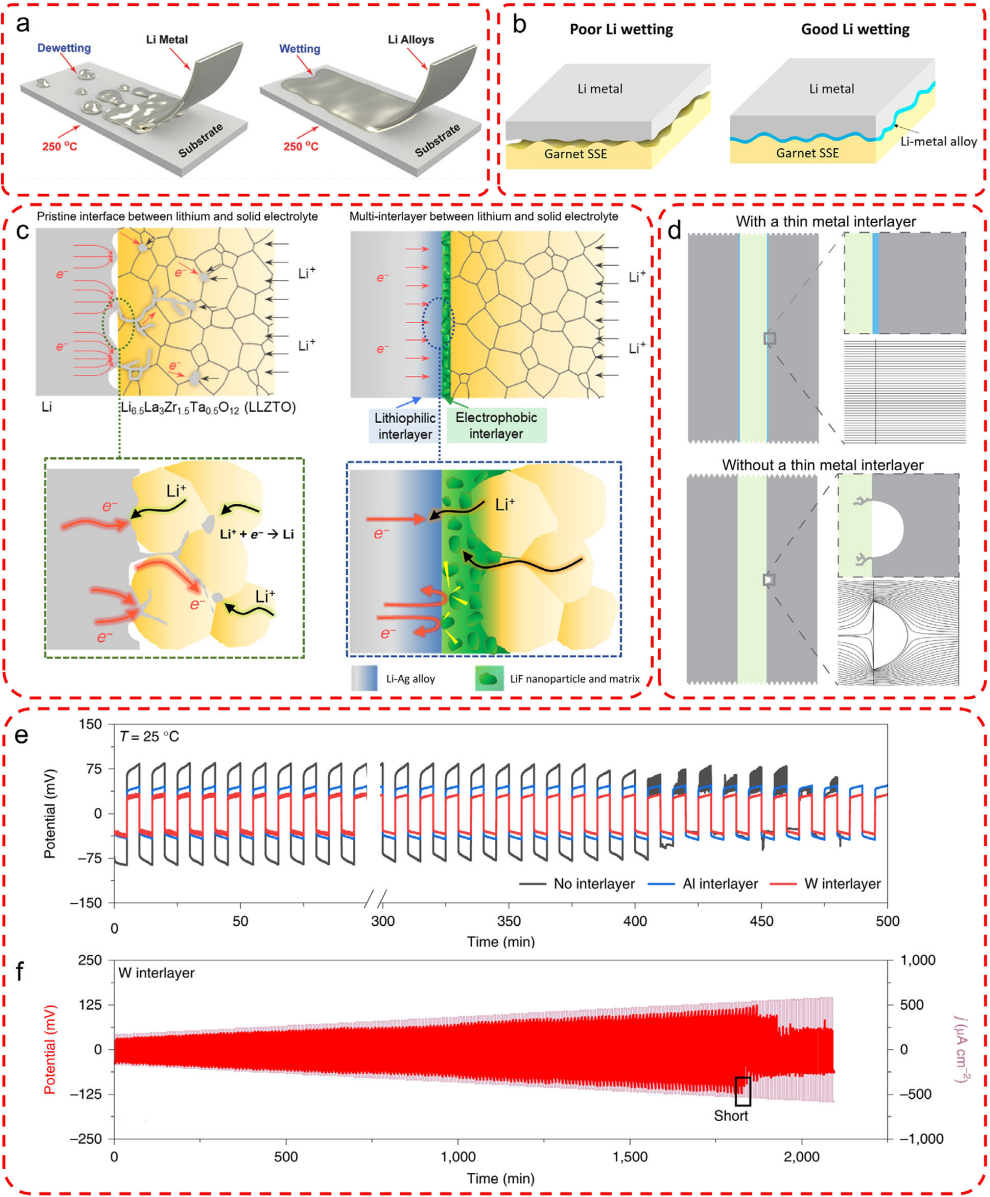
(a) Schematic of soldering Li and Li alloys onto substrates, illustrating poor wetting of pure molten Li on solid substrates compared to the improved contact achieved with Li alloys. (b) Schematic of an engineered garnet SSE|Li interface, showing how an Al coating forms a lithiophilic Li‐Al alloy, improving contact and reducing the area‐specific resistance. (c) Schematic illustrations of the Li metal and garnet‐type solid electrolyte interface, comparing dendrite formation at a conventional interface with an improved design incorporating lithiophilic and electron‐blocking interlayers, alongside enlarged views of each interface. (d) Schematic illustrating the interfacial differences in symmetric cells: a Li|IL|SSE|IL|Li cell achieves continuous contact for uniform current distribution, whereas a Li|SSE|Li cell with discontinuous contact concentrates current density at void edges. (e) Galvanostatic Li plating and stripping curves at 100 µA cm^−2^ for Li|LLZTO|Li, Li|Al|LLZTO|Al|Li, and Li|W|LLZTO|W|Li symmetric cells show lower overpotential in cells with ILs and (f) cycling stability of Li|W|LLZTO|W|Li symmetric cells. (a) Reproduced with permission [94]. Copyright 2017, Wiley‐VCH. (b) Reproduced with permission [122]. Copyright 2017, AAAS. (c) Reproduced with permission [128]. Copyright 2022, AAAS. (d–f) Reproduced with permission [59]. Copyright 2022, Springer Nature.

Critically, the Li‐Sn alloy mitigates the interfacial contact issue with one of the most promising SSEs (garnet‐type electrolytes, such as LLZO). Garnet SSEs generally suffer from poor interfacial contact with Li, resulting in high interface resistance and inhomogeneous current distribution. The intrinsic poor wettability of Li metal on garnet surfaces is evident from contact angles of approximately 90 to 120 degrees [136]. The use of molten Li–Sn alloy lowers the interfacial energy mismatch with garnet particles, thereby improving the wettability of the garnet surface by the Li metal anode (Figure 13a) [94].

In addition, Li alloys can serve as interfacial protective layers to further improve Li|garnet contact. For example, a thin Li–Al alloy layer can fill interfacial voids between the Li metal and SSE, thereby increasing the effective contact area and reducing the interfacial resistance (Figure 13b). The resistance drops from 950 Ω cm^2^ at room temperature (20°C) to 75 Ω cm^2^, and further decreases to 30 Ω cm^2^ at 60°C [122].

Building upon these efforts, a multilayer interfacial architecture comprising a lithiophilic Li–Ag alloy layer and an electrically insulating LiF layer for electron blocking has recently been developed (Figure 13c). This configuration reduces the interfacial resistance to 13.4 Ω cm^2^ at 60°C. Compared with a single lithiophilic alloy layer, the multilayer structure effectively prevents electron leakage from the anode to the electrolyte under elevated temperature (60°C) and high electric field (3 V), while maintaining low electronic conductivity [128].

Furthermore, the introduction of a thin metal interlayer directly between Li metal and the SSE has emerged as an efficient approach to mitigate interfacial impedance and maintain continuous contact, thereby avoiding void formation (Figure 13d) [59, 137]. Several thin metals have been employed to enhance the critical current density by forming stable Li|SSE interfaces. Among them, tungsten (W) is particularly effective, providing high thermodynamic and kinetic barriers against Li‐void accumulation. This property minimizes void formation and promotes void dissolution across most surface orientations (Figure 13e) [59]. Consequently, the W interlayer stabilizes the Li anode morphology and enhances cycling stability (Figure 13f), rendering it a promising approach for ultrathin Li anodes.

Notably, interfacial voids (recognized as precursors to Li dendrite nucleation and growth) typically form before dendrite propagation at the Li|garnet interface [58, 59]. Therefore, when void formation is effectively suppressed, Li deposition occurs uniformly within the SSE. Nevertheless, while employing thin metal interlayers significantly improves interfacial contact, the introduction of additional interlayer materials also complicates efforts to minimize anode thickness, which remains a critical challenge for practical ultrathin Li metal designs.

**FIGURE 14:**
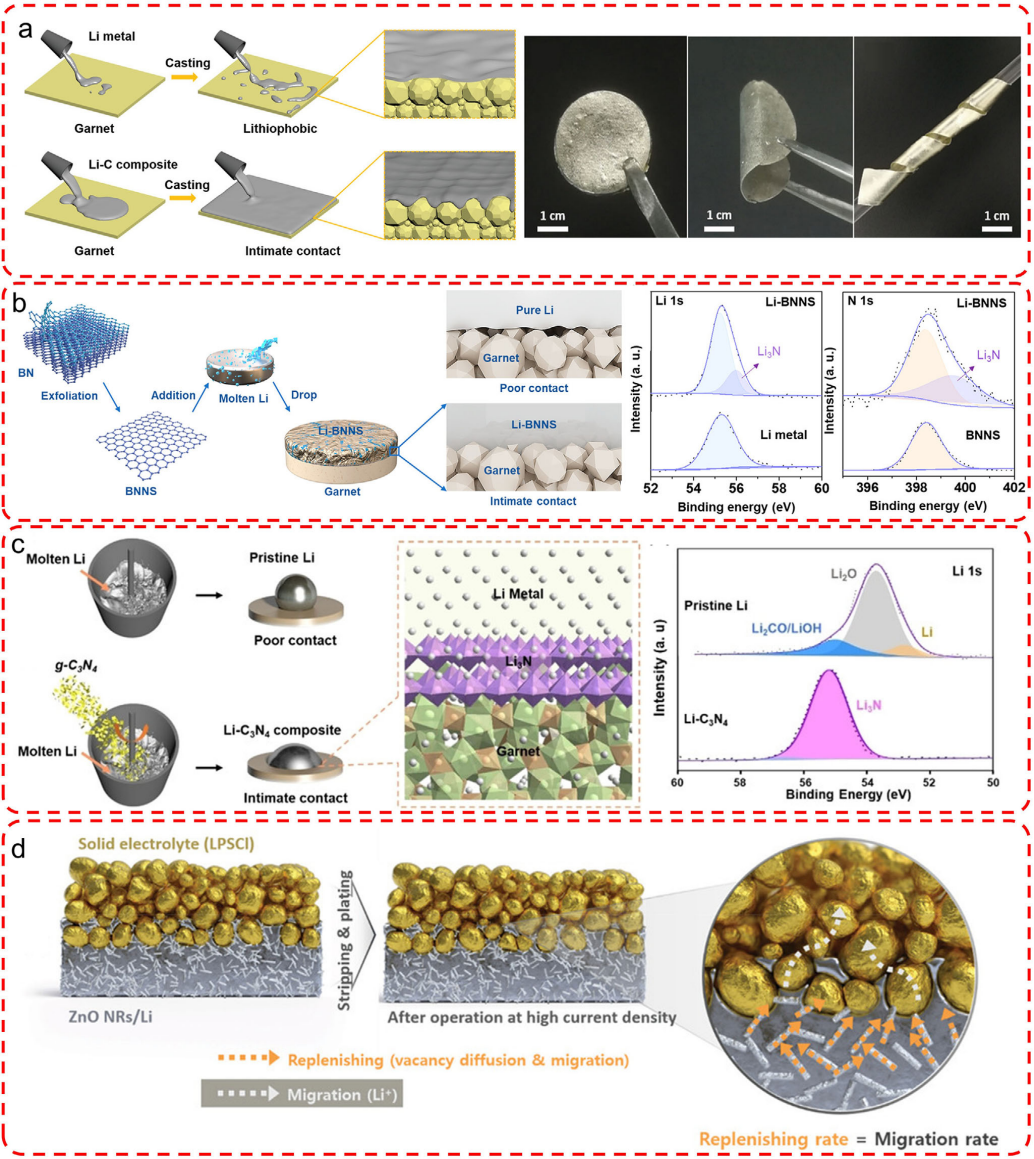
(a) Schematic showing poor contact of pure Li with garnet SSEs, while the paste‐like lithium–graphite (Li–C) composite spreads well, forming intimate contact with metallic luster, flexibility, and twistability. (b) Schematic of boron nitride nanosheets (BNNS) and Li‐ BNNS preparation, highlighting Li‐BNNS's superior contact with garnet and its Li 1s and N 1s XPS characterization. (c) Li‐C_3_N_4_ forms intimate contact with garnet, creating an in situ Li3N layer for low resistance and stable cycling, confirmed by Li 1s XPS analysis. (d) ZnO NRs/Li promotes fast Li‐ion transport, preventing voids and dendrites at the Li|SE interface. (a) Reproduced with permission [138]. Copyright 2019, Wiley‐VCH. (b) Reproduced with permission [139]. Copyright 2019, American Chemical Society.(c) Reproduced with permission [140]. Copyright 2020, Wiley‐VCH. (d) Reproduced with permission [141]. Copyright 2021, Wiley‐VCH.

The second strategy involves introducing a buffer layer between the Li metal and the garnet SSEs to improve wettability. This buffer layer can enhance interfacial contact and fill voids [142, 143]. However, adding an extra buffer layer inevitably increases the thickness of the Li metal anode, which may be contradictory to the concept of using ultrathin Li. Adding the buffer material directly to molten Li metal allows the convenient preparation of ultrathin Li via coating methods, while the added buffer substance performs its intended role. For instance, Huang's team infused chemically lithiated graphite powder into molten Li, leading to its spontaneous uniform dispersion within the Li (Figure 14a). This created a Li‐C composite material with tunable fluidity and viscosity, which could be cast onto the garnet SSE like a paste, and achieving close contact [138]. However, the conductivity of the lithiated graphite powder presented a new challenge at the interface. To address this, they introduced hexagonal boron nitride (h‐BN), an electrical insulator, as a graphite analogue, resulting in a Li metal‐boron nitride nanosheet material with high adhesiveness that adheres well to the garnet SSE (Figure 14b) [139]. The boron nitride nanosheets (BNNS) at the interface could foster the formation of a Li_3_N layer, similar to the capability of another material they reported, graphitic carbon nitride (g‐C_3_N_4_). The g‐C_3_N_4_ interacts with Li to form a Li_3_N layer that is beneficial for Li‐ion conduction but not electrically conductive (Figure 14c), effectively isolating the garnet from the electronic contact of Li metal, and suppressing dendrite formation without affecting Li‐ion conductivity [140].

Moreover, there exists a rate disparity between the migration of Li ions to the cathode at the interface and the replenishment of Li toward the interface from within the Li metal, especially under high current, where the latter is much slower, leading to severe void formation between the SSE and Li metal. This issue can be addressed by combining lithiated zinc oxide nanorods (ZnO NRs) with molten Li metal to improve the rate of Li supply to the interface from the Li metal (Figure 14d). Due to the Li‐ion conductivity of the NRs, they provide a channel for Li transport from the bulk Li to the Li|SSE interface, thereby enhancing the replenishment rate, avoiding void formation, and inhibiting dendrite growth [141].

### 5.4 Design Principles of Ultrathin Li Metal|Electrolyte Interface

Rational interfacial engineering through electrolyte additives or the direct introduction of artificial interphases is indispensable to enable ultrathin Li metal, given their inherently limited lithium reservoir. An ideal interfacial layer on Li metal should combine high ionic conductivity with negligible electronic conductivity, while possessing sufficient mechanical conformability and robustness to accommodate the substantial interfacial stress fluctuations during Li plating/stripping. In ASSLMBs, particularly those employing inorganic solid‐state electrolytes, a nanoporous interfacial architecture is further required to alleviate stress accumulation and preserve intimate solid‐solid contact. The following aspects should receive greater attention in future research on the interface engineering of ultrathin Li metal.

1. Developing low‐cost, scalable, and compatible with roll‐to‐roll processing strategies for interphase engineering is essential to enable the practical usage of ultrathin Li metal. In this regard, blade‐coating and vapor deposition with high‐throughput approaches are likely to be more economically viable than other complex and costly fabrication methods.
2. The thickness of the interface layer should be precisely controlled to ensure fast interfacial charge transfer and match the thickness of Li metal (i.e., ≤100 nm), and minimize the gravimetric/volumetric energy density sacrifice of Li metal. Moreover, emphasis should be placed on cell operation performance under practical conditions, including lean electrolyte (≤ 3 g Ah^−1^) and high areal capacity (≥ 3 mAh cm^−2^). Moreover, the cycling performance of Ah‐level (i.e., ≥ 5 Ah) pouch cells should also be demonstrated to validate the practical viability of ultrathin Li metal.
3. The integration of fundamental studies with advanced analytical techniques should be further reinforced to accelerate the development of ultrathin Li metal. In particular, operando cryo‐transmission electron microscopy, atomic force microscopy, and synchrotron‐based characterization are indispensable for elucidating SEI evolution, Li nucleation and growth dynamics, as well as the underlying failure mechanisms in ultrathin Li metal.

## 6 Current Collectors for Ultrathin Li Metal

### 6.1 Challenges of Current Collectors in Ultrathin Li Metal

We have previously discussed that reducing the thickness of Li metal anodes brings multiple benefits, among which one of the most important is the enhancement of energy density. As an indispensable yet electrochemically inactive component in batteries, the current collector, when paired with ultrathin Li anodes, can significantly influence the overall cell performance—including energy density, rate capability, and long‐term stability—through both its intrinsic density and its ability to regulate Li deposition morphology [144]. The solid–solid interface between Li metal and the current collector plays a critical role in determining battery performance. Therefore, in practical applications, especially under limited Li inventory, the material properties and geometric structure of current collectors supporting ultrathin Li must be comprehensively considered in battery design. Conventional commercial current collectors are primarily based on copper and aluminum foils. Continuous efforts have been devoted to reducing their thickness from ∼20 to ∼6 µm, accompanied by a gradual decrease in their weight fraction (from ∼18% in 2000 to ∼6% in 2025) [145, 146, 147]. At present, a wide range of materials (e.g., aluminum, copper, stainless steel, carbon‐based materials, and composites) and structural designs (e.g., foil‐type, mesh, porous, and coated configurations) have been developed [148].

**FIGURE 15:**
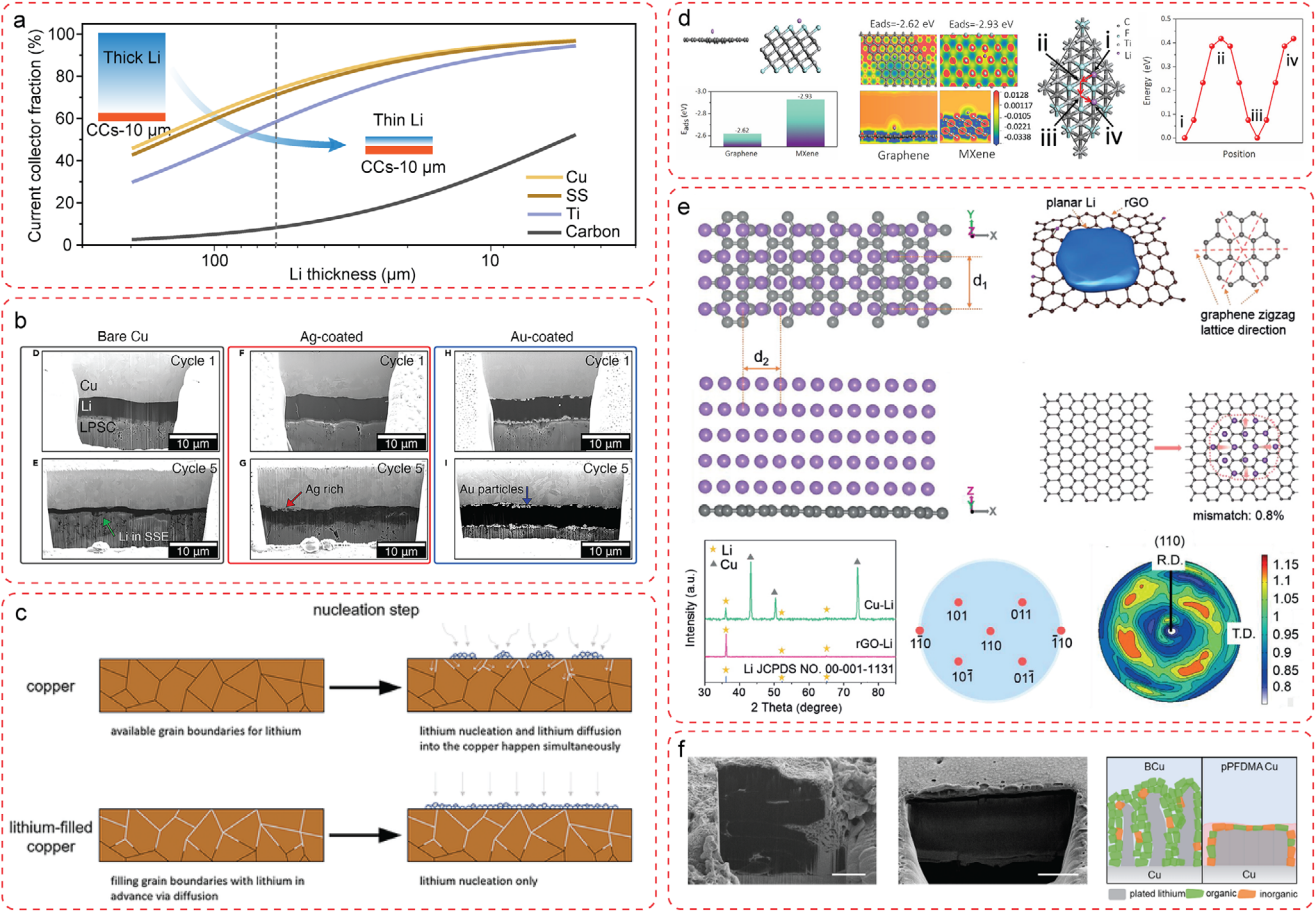
(a) Current collector mass fraction as a function of lithium thickness for Cu, stainless steel (SS), Ti, and carbon. (b) Cryo‐FIB‐SEM images of Li deposited onto bare Cu, Ag‐modified Cu, and Au‐modified Cu electrodes after the first and fifth deposition cycles. (c) Schematic illustrations of the different lithium nucleation behaviors on copper and prelithiated copper, respectively. (d) Mechanistic illustration of the MG film for Li deposition, including Li binding energies on graphene and MXene, charge density difference at the Li adsorption site, and migration pathways with the corresponding diffusion energy barriers for Li diffusion on MXene. (e) Atomic configuration, growth behavior, and crystallographic characteristics of Li on the rGO substrate. Optimized structures (plan and side views) and schematic illustrations of Li on rGO are shown, followed by XRD patterns of standard Li, Li on Cu, and Li on rGO, together with Li (110) plane projection and orientation analysis. (f) Cryo‐FIB–SEM comparison shows that Li deposition on the pPFDMA‐coated Cu surface results in a dense and uniform morphology, in contrast to the porous dendritic structure observed on bare Cu. (b) Reproduced with permission [150]. Copyright 2023, Elsevier. (c) Reproduced with permission [151]. Copyright 2023, Wiley‐VCH. (d) Reproduced with permission [152]. Copyright 2019, American Chemical Society. (e) Reproduced with permission [153]. Copyright 2019, Wiley‐VCH. (f) Reproduced with permission [154]. Copyright 2026, Elsevier.

In general, current collectors are required to exhibit high electrical conductivity to minimize internal resistance and mitigate thermal risks under high‐rate operation; good electrochemical stability, particularly across wide potential windows, to ensure compatibility with the electrolyte; and sufficient mechanical strength to maintain electrode integrity. For soft Li foils, especially ultrathin Li, the mechanical strength and flexibility of the current collector serve as essential structural support [149]. In addition, low density and reduced thickness are critical for minimizing the fraction of inactive components and maximizing overall energy density, while low cost and recyclability remain important considerations from both economic and environmental perspectives. As shown in Figure 15a, when paired with thick Li, mainstream metallic foil current collectors, such as copper, stainless steel, and titanium, already account for more than 30% of the total mass in composite Li anodes. As the Li thickness decreases, the mass fraction of high‐density copper foil increases markedly (reaching ∼92% when paired with 15 µm Li), whereas lightweight materials such as carbon‐based collectors maintain a significantly lower fraction (∼26% under the same conditions). Therefore, when matching with ultrathin Li, greater attention should be directed toward the impact of current collector density on the overall energy density of the battery.

When paired with ultrathin Li anodes, or even in anode‐free configurations, Li is directly deposited onto the current collector. As a result, both the material properties and the topological structure of the current collector critically influence Li nucleation and growth behavior at the interface. Irreversible Li deposition leads to rapid Li consumption, particularly under limited Li inventory. In addition, certain current collector designs or modification strategies (e.g., thick 3D carbon frameworks) may introduce excessive electrode thickness, while others (e.g., molten Li infusion) may result in uncontrolled Li utilization, posing challenges for practical applications of ultrathin Li anodes. Therefore, rational modification strategies and geometric design of current collectors are essential to precisely regulate the solid–solid Li–current collector interface, control Li deposition behavior, suppress dead Li formation, and ultimately improve the cycling stability of ultrathin Li anodes.

### 6.2 Foil‐Type Current Collectors

Foil‐type current collectors are thin and compatible with roll‐to‐roll processing. Although commercial aluminum foil is widely used as a cathode current collector due to its low weight and cost, its application on the anode side remains challenging because Li can alloy with aluminum, leading to severe volume expansion and structural pulverization [155, 156]. Currently, copper, nickel, titanium, stainless steel, and carbon‐based materials are the mainstream choices for Li metal anode current collectors [146, 157]. Beyond conventional metallic foils and carbon materials, emerging materials such as graphene, MXene, and metal–organic frameworks (MOFs) have also been introduced, offering advantages in density, electrochemical stability, thermal conductivity, and flexibility [158]. Unlike metallic foils, which are typically thinned via rolling processes, these two‐dimensional materials can be fabricated into films with thicknesses comparable to metallic foils (∼10 µm or even thinner) through simple self‐assembly methods. When paired with ultrathin Li anodes, foil‐type current collectors often rely on their intrinsic properties to regulate Li deposition behavior at the interface. However, inherent limitations still exist for different types of foil current collectors, and therefore, surface modification is generally required. Various coating strategies have been developed for surface modification, including electrochemical methods (e.g., electrodeposition, electrochemical prelithiation, and pulsed nucleation treatments), in situ reactions (e.g., oxidation, etching, carbonization, and reduction), and vapor‐phase deposition techniques (e.g., magnetron sputtering, atomic layer deposition (ALD), thermal evaporation, and chemical vapor deposition (CVD), including low‐pressure chemical vapor deposition (LPCVD) and plasma‐enhanced chemical vapor deposition (PECVD)) [159, 160, 161]. When applied to ultrathin Li systems, it is important to consider whether these coating processes introduce additional thickness. Vapor‐phase techniques can produce ultrathin coatings with precisely controlled thickness, enabling effective surface modification without significantly increasing the overall thickness. In contrast, methods such as slurry coating or doctor‐blade casting are less suitable for forming ultrathin modification layers and may lead to increased thickness and mass of the current collector [162].

During charging, Li ions migrate toward the current collector surface under the electric field, where they are reduced to form Li nuclei, followed by surface diffusion and subsequent growth. Therefore, the binding energy between Li and the current collector, as well as the migration energy barrier on the substrate surface, plays a critical role in Li nucleation and early‐stage growth. The concept of lithiophilicity is commonly used to describe the affinity between Li and the current collector [163]. Copper foil plays a crucial role as a current collector in Li batteries and has been widely commercialized due to its compatibility with roll‐to‐roll manufacturing. However, copper tends to induce non‐uniform Li deposition, leading to dendritic growth and the formation of porous Li structures [164]. Continuous efforts have been devoted to constructing lithiophilic coatings or nanostructures on copper surfaces to promote uniform Li nucleation and deposition. A variety of materials, including metals, metal compounds, and polar functional groups, have been explored as lithiophilic coatings to enhance the affinity of the substrate. As discussed above, strategies to improve the lithiophilicity of current collectors have also been widely employed in the fabrication of ultrathin Li metal anodes. Metals such as Ag, Au, Al, Zn, and In are commonly used due to their ability to form Li alloys, thereby reducing nucleation barriers. However, most Li alloy phases primarily regulate Li nucleation at the initial stage, and their guiding effect gradually diminishes during subsequent deposition. In addition, repeated alloying/dealloying processes may lead to structural degradation or detachment of the modification layer [165]. From a thermodynamic perspective, Li‐rich solid solution alloys (e.g., Li–Ag) exhibit lower phase transformation barriers and better reversibility, whereas intermetallic compounds with fixed stoichiometry (e.g., Li–Al and Li–Zn) suffer from limited reversibility due to higher structural transformation barriers [166]. Notably, such lithiophilicity engineering strategies are effective not only in liquid electrolyte systems but also in solid‐state batteries. Stephanie Elizabeth Sandoval et al. studied the structural evolution of lithiophilic Ag and Au alloy modification layers in sulfide solid‐state batteries using synchrotron X‐ray micro‐computed tomography and cryogenic/plasma focused ion beam (FIB) techniques [150]. The results indicate that the alloy layers promote uniform Li nucleation and growth. During cycling, the alloy layers break into particles and disperse throughout the Li layer, thereby improving the stability of solid–solid interfacial contact (Figure 15b). Compared with introducing lithiophilic coatings via complex processes, pre‐treatment of copper foils provides a more cost‐effective and scalable approach. It has been reported that Li may diffuse into the copper substrate along grain boundaries during the nucleation stage, resulting in a reduced number of surface nuclei and non‐uniform deposition (Figure 15c) [151]. By applying short pulsed potentiostatic nucleation treatments (e.g., 10 ms at −1.5 V), a higher nucleation density can be achieved, leading to more compact and uniform Li deposition. Importantly, this strategy does not introduce additional coating thickness, making it particularly suitable for ultrathin Li systems.

The above lithiophilicity enhancement strategies can also be applied to carbon‐based current collectors. In particular, heteroatom‐doped carbon materials provide abundant functional groups, offering more diverse approaches for introducing lithiophilic sites. Such strategies based on intrinsic carbon chemistry can effectively minimize additional thickness while improving interfacial affinity. Moreover, carbon layers can also be used as lithiophilic coatings on metallic current collectors. For example, porous carbon networks with nitrogen (N) and sulfur (S) co‐doping have been constructed on commercial copper foils via simple carbonization processes, serving as lithiophilic sites to enable uniform Li deposition [167]. In addition, emerging materials such as MXene, which possess intrinsically polar surface terminations, exhibit strong lithiophilicity and can effectively induce uniform Li nucleation [168]. It has been reported that the binding energy of Li on MXene surfaces (−2.93 eV) is higher than that on graphene (−2.62 eV), indicating stronger Li affinity (Figure 15d) [152]. Furthermore, the high local charge density between MXene and Li atoms suggests stronger interactions during Li anchoring. Defects in MXene (e.g., Ti vacancies) can also reduce Li migration barriers and significantly accelerate Li diffusion. These emerging two‐dimensional materials can be fabricated into large‐area, ultrathin current collectors through scalable self‐assembly processes [169]. However, although enhancing lithiophilicity is an effective strategy to suppress dendrite formation, most modification layers primarily influence Li nucleation at the initial stage. For ultrathin Li anodes, this may be sufficient in early cycles; however, long‐term effectiveness remains questionable due to possible degradation or detachment of the modification layer during repeated cycling. While Li–Ag solid‐solution alloys offer improved reversibility, the use of noble metals inevitably increases material costs. Therefore, when designing lithiophilic coatings, the interfacial stability between the coating and the current collector substrate must be carefully considered.

The deposition of metallic Li is essentially an electrochemical crystallization process occurring on the current collector surface. For Li crystals with a body‐centered cubic (BCC) structure, the surface migration and nucleation of Li atoms are strongly influenced by the crystallographic orientation of the current collector. As discussed above, the Cu(100) facet is more favorable for forming low‐energy crystallographic orientation relationships with the close‐packed Li(110) plane, which can facilitate lateral Li growth relative to the substrate. From the perspective of Wulff construction, such a lateral growth mode is beneficial for achieving dense Li deposition [144]. A dense Li morphology can effectively reduce side reactions with the electrolyte, suppress dead Li formation, and improve the cycling stability of ultrathin Li metal anodes. Crystal orientation engineering therefore enables the regulation of Li deposition without introducing additional coating thickness, making it particularly attractive for ultrathin Li systems. However, the large‐scale fabrication of single‐crystal metallic foils remains challenging. As an alternative, carbon‐based materials have attracted increasing attention for regulating Li deposition orientation. For example, Li et al. employed a 23 µm self‐assembled reduced graphene oxide (rGO) substrate as the current collector for ultrathin Li metal anodes, achieving epitaxial Li growth along the Li(110) plane on the rGO film (Figure 15e) [153]. Notably, at an areal capacity of 5 mAh cm^−2^, the thickness of the deposited Li layer was only 25.3 µm, comparable to that of Li foil. By matching such a lightweight current collector with ultrathin Li, a high energy density of up to 754 Wh kg^−1^ was achieved in flexible Li–sulfur battery systems. In addition to the interaction between Li and the current collector, the interaction between the current collector and the electrolyte is also of critical importance. By regulating the local solvation environment at the interface, side reactions can be suppressed, thereby reducing Li consumption and dead Li formation. For example, an ultrathin solvent‐ and electrolyte‐repellent coating (∼15 nm) has been constructed on copper current collectors via initiated chemical vapor deposition (iCVD), effectively suppressing electrolyte decomposition (Figure 15f) [154]. This approach enables the formation of a thin, inorganic‐rich SEI dominated by selective salt reduction, without compromising energy density, and is therefore well suited for anode‐free Li metal battery systems.

### 6.3 Three‐Dimensional Current Collectors

For thin Li metal anodes, a low N/P ratio implies significant volume variation of the Li anode during cycling, posing severe challenges to interfacial stability. The introduction of three‐dimensional (3D) current collectors has played an important role in mitigating volume expansion and reducing local current density [170]. However, 3D current collectors often lead to increased electrode thickness, thereby compromising volumetric energy density. Therefore, when designing 3D current collectors for ultrathin Li anodes, particular attention should be paid to the balance between capacity and thickness. Most currently reported 3D current collectors typically exhibit high mass, especially for metallic frameworks, which inevitably increases the fraction of inactive components, thereby offsetting the energy density advantages brought by ultrathin Li anodes. Therefore, for 3D current collectors paired with ultrathin Li, not only their ability to regulate Li deposition but also their intrinsic density and thickness are of critical importance. To address these challenges, various advanced fabrication strategies have been developed to construct lightweight and thin 3D current collectors with tailored architectures, including self‐assembly, template‐assisted methods, electrospinning, and 3D printing. The resulting micro‐/nanostructures can effectively accommodate the volume variation of Li metal and facilitate rapid ion transport [158, 171].

**FIGURE 16:**
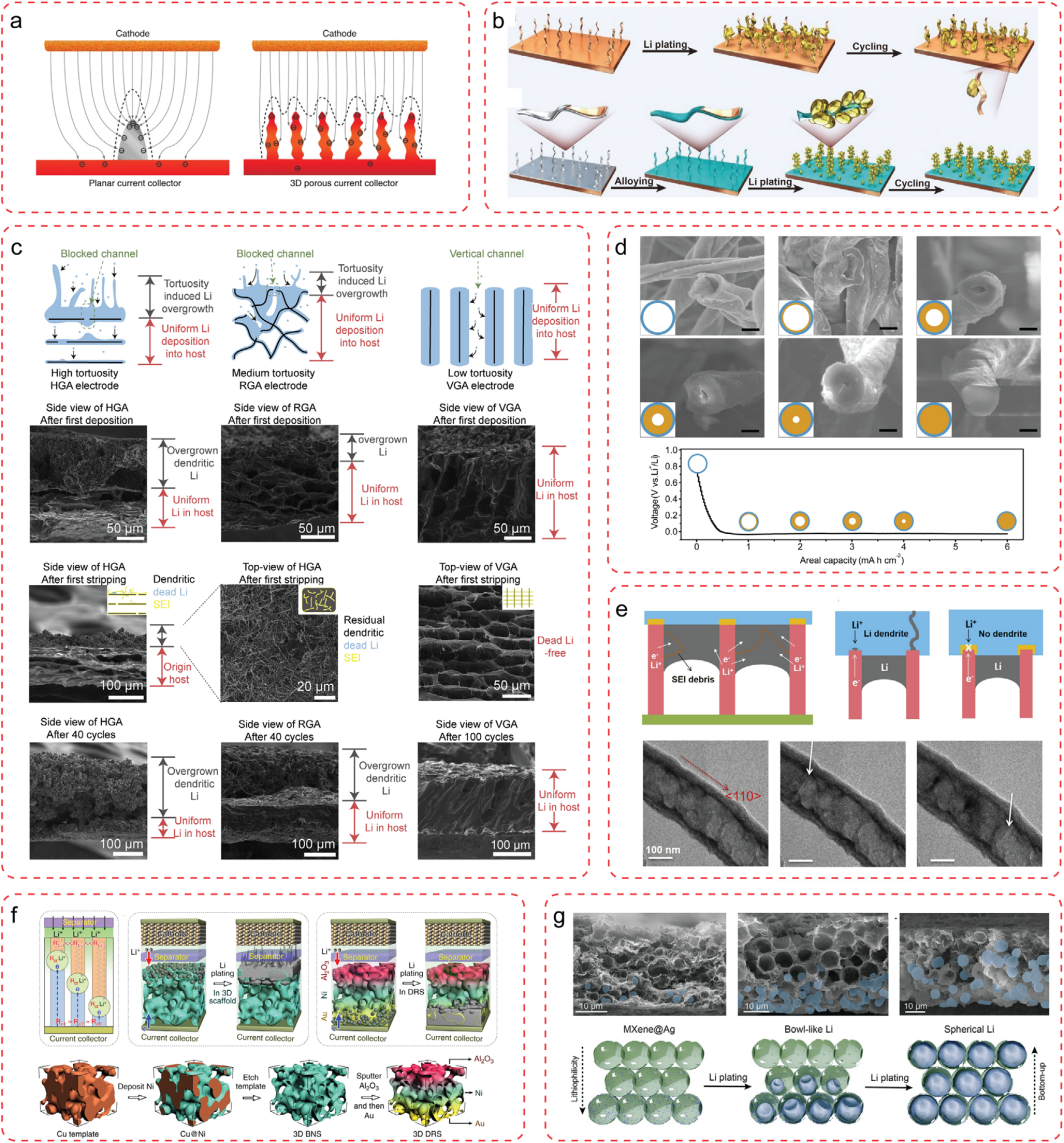
(a) Illustration of the proposed electrochemical deposition processes of Li metal on a planar current collector and a 3D current collector. (b)Schematic of the Li plating process on the (a) 3D Cu foil and (b) 3D Cu@Al foil. (c) Li deposition and cycling behavior in HGA, RGA, and VGA anodes. SEM images show the morphology after initial deposition, subsequent stripping, and extended cycling (40 and 100 cycles). Cycling was performed at 3 mA cm^−2^ with a capacity of 3 mAh cm^−2^ in a carbonate electrolyte. (d) Cross‐sectional SEM images showing Li deposition behavior in 3D‐HCFs at increasing areal capacities (1–6 mAh cm^−2^), together with a schematic illustration of the corresponding structural evolution. Scale bars, 5 µm. (e) SEI debris within the tubules does not disrupt ionic and electronic percolation. The ELI component serves as an inert binder between SE and MIEC, suppressing dendrite formation. TEM images show single‐crystalline Li confined within the carbon tubules. (f) Top‐ and bottom‐up Li growth modes and the DRS fabrication route, including transport and charge‐transfer resistances across the anode thickness, top‐growth at the anode/separator interface, bottom‐up deposition in a DRS anode, and the fabrication process. (g) Side‐view SEM images of Li deposition on the MXene@Ag membrane, together with a schematic illustration of the spherical Li plating behavior. (a) Reproduced with permission [172]. Copyright 2015, Springer Nature. (b) Reproduced with permission [173]. Copyright 2018, Wiley‐VCH. (c) Reproduced with permission [174]. Copyright 2020, Elsevier. (d) Reproduced with permission [175]. Copyright 2017, Elsevier. (e) Reproduced with permission [177]. Copyright 2020, Elsevier. (f) Reproduced with permission [179]. Copyright 2019, Springer Nature. (g) Reproduced with permission [180]. Copyright 2023, Wiley‐VCH.

Compared with foil‐type current collectors, 3D architectures provide additional space accommodating the large volume expansion of Li metal. Moreover, their high specific surface area can effectively reduce local current density and guide uniform Li deposition. For example, transforming commercial planar copper foil into a three‐dimensional porous copper structure increases the pore area per unit geometric area to ∼45 cm^2^ cm^−2^, leading to a more uniform electric field distribution and homogeneous charge transport along the copper framework (Figure 16a) [172]. It is worth noting that 3D current collectors should possess an appropriate pore size. Excessively large pores weaken the spatial confinement effect, causing Li to detach from the 3D framework and lose electrical contact during long‐term cycling, thereby leading to the formation of dead Li [172]. Therefore, beyond optimizing the pore structure, lithiophilic modification is typically introduced to enhance the interaction between Li and the current collector, improving Li hosting capability and interfacial stability, while suppressing dead Li formation within large pores. For instance, in situ electrochemical formation of lithiophilic binary Li–Al alloy layers on 3D frameworks can guide uniform Li nucleation and growth (Figure 16b) [173]. Meanwhile, the Li–Al alloy can serve as a Li reservoir to compensate for irreversible Li loss during cycling, thereby enabling improved long‐term stability of composite Li metal.

In addition to high surface area and porosity, an appropriate geometric design provides a new perspective for regulating Li deposition behavior. In the development of gradient‐designed 3D current collectors, tortuosity plays a crucial role in Li deposition. Chen et al. constructed reduced graphene oxide (rGO) electrodes with vertically aligned, horizontally aligned, and randomly arranged structures, corresponding to tortuosities of 1.25, 4.46, and 1.76, respectively, to investigate the effect of tortuosity on Li deposition behavior (Figure 16c) [174]. It was found that increasing tortuosity leads to a reduction in effective internal surface area and an increase in local current density at the electrode top (Figure 16c), thereby promoting excessive dendritic Li growth and dead Li formation in the upper region. Therefore, low‐tortuosity and vertically aligned 3D current collectors are more favorable for reversible Li cycling. To further improve space utilization and reduce ineffective voids, Liu et al. designed a 3D carbon fiber framework with large free space, hollow structures, and high electroactive surface area [175]. Unlike uncontrolled large pores, the hollow architecture offers confined and accessible internal space, which enables efficient Li accommodation while suppressing the formation of electrically isolated Li.This framework can theoretically accommodate up to 9.77 mg cm^−2^ of Li, enabling the design of ultrathin composite Li anodes. The hollow structure increases the effective pore volume (1.83 × 10^−2^ cm^3^ cm^−2^), while appropriate pore sizes induce preferential Li growth within the internal cavities (Figure 16d). This not only suppresses dendrite formation but also reduces electrode‐level volume change and potential safety risks.

In solid‐state batteries, the utilization of pore space has also been demonstrated as an effective strategy to regulate the Li deposition behavior and accommodate the electrochemically generated significant mechanical stress. Electrochemically induced mechanical stress can deteriorate solid–solid interfacial contact and lead to cracking of the solid electrolyte (Figure 16e) [176]. Moreover, unlike liquid electrolytes, solid electrolytes cannot readily maintain contact with dynamically evolving Li during deposition. This challenge becomes even more severe in ultrathin Li or anode‐free systems, where Li volume change is effectively unbounded [177]. To address this issue, Chen et al. designed a 3D architecture composed of mixed ionic–electronic conductors (MIECs) and electronic and Li‐ion insulators (ELIs) (Figure 16e) [121]. The MIEC/ELI‐mediated interfaces provide robust electron and ion transport pathways, while the confined pore space regulates Li plating and stripping. To enable diffusion‐controlled creep mechanisms such as Nabarro–Herring creep, pore size plays a critical role. A critical size range of ∼200–500 nm was proposed, within which stress can be maintained at relatively low levels while allowing high current density operation. When the pore size exceeds ∼500 nm, dislocation‐based creep (power‐law creep) may dominate, leading to significantly increased stress that can compromise the mechanical integrity of solid‐state batteries.

Beyond geometric optimization, heterogeneous 3D current collectors provide additional opportunities for regulating Li deposition. Li deposition is a diffusion‐coupled process, and in heterogeneous structures, the deposition location and rate are governed by multiphysical parameters, including Li‐ion concentration, local potential, and current density. Rational gradient design can effectively induce a “bottom‐up” Li deposition mode within 3D architectures [178]. This deposition behavior enables full utilization of internal space and confines Li growth within the host structure. Various gradient strategies have been developed, including conductivity gradients, lithiophilicity gradients, dual ionic/electronic gradients, and pore‐size gradients, which can be achieved through doping/defect engineering, electric field regulation, external field application, and tailored SEI chemistry [178]. It has been demonstrated that Li preferentially nucleates on substrates with lower nucleation overpotential or energy barriers (e.g., Au, Pt, Sn, and ZnO), providing thermodynamic guidance for deposition site regulation. To suppress preferential Li growth at the top region (near the separator), Pu et al. designed a 3D current collector with combined conductivity and lithiophilicity gradients [179]. The insulating top layer blocks electron transport at the anode/separator interface, while the lithiophilic bottom layer (e.g., Au coating) reduces nucleation barriers (Figure 16f). As a result, a bottom‐up Li deposition behavior is achieved through the synergistic effect of conductivity and lithiophilicity gradients. However, most gradient designs require relatively thick 3D structures (typically 50–100 µm or even thicker) to establish sufficient gradient differences, which increases both thickness and mass, making them less compatible with ultrathin Li systems. By combining lithiophilicity gradients with pore‐size regulation, it has been demonstrated that ordered bottom‐up deposition of spherical Li can be achieved within a thickness of ∼25 µm (Figure 16g) [180]. In such systems, diluted Ag nanoparticles are used to construct a continuous lithiophilicity gradient, while curvature optimization of the framework enables Li growth within internal pores. The resulting spherical Li morphology significantly reduces interfacial side reactions and suppresses dead Li formation, enabling long‐term cycling stability (>3000 h) and high Li utilization (>90%).

Although 3D current collector strategies have shown promising performance in liquid or quasi‐solid systems, their application in all‐solid‐state batteries remains challenging. The complex 3D structures introduce difficulties in maintaining intimate solid–solid interfacial contact and efficient electron/ion transport. Therefore, further efforts are required to design suitable geometries that ensure continuous ion and electron pathways at solid–solid interfaces.

## 7 Summary and Outlook

Developing practical high‐energy LMBs with commercial viability, all cell parameters require strict control, particularly the thickness of the Li metal anode. Using ultrathin Li metal (≤15 µm) provides a direct route to high energy density while minimizing material consumption, and offers a more stringent and representative platform for evaluating strategies to extend cycle life and enhance Coulombic efficiency.

**FIGURE 17:**
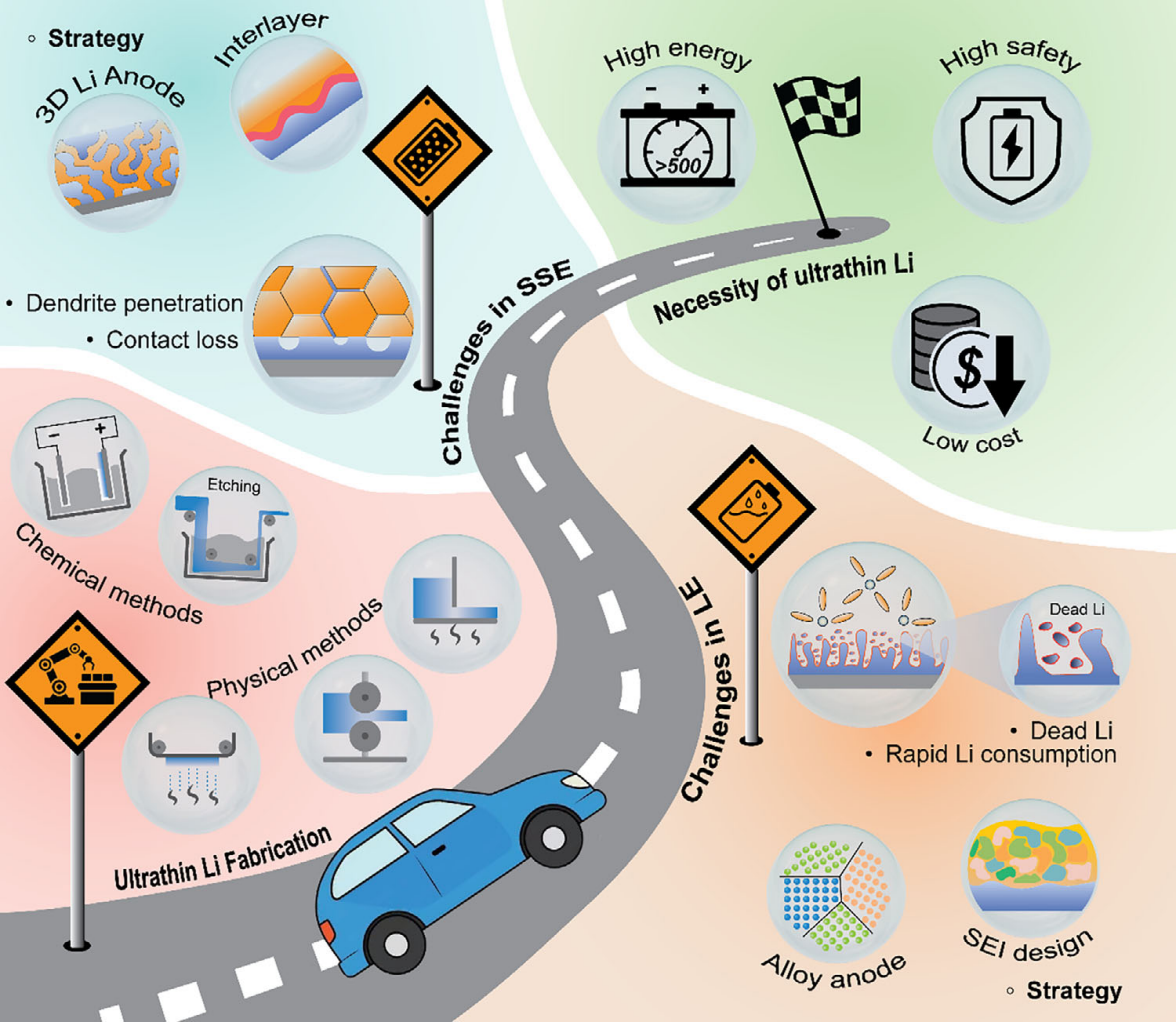
Fabrication and practical challenges in industrial applications of ultrathin Li anodes.

Motivated by the advantages of ultrathin Li, a variety of chemical and physical methods have been developed for its fabrication (Figure 17). Electrodeposition is the most widely reported chemical approach for controlling the thickness of deposited lithium metal; however, it typically yields porous, mossy morphologies that fall short of practical application requirements. Moreover, the reliance on flammable and toxic organic electrolytes raises significant safety and environmental concerns. By contrast, physical approaches to fabricating ultrathin lithium metal are more promising. Among these approaches, mechanical calendaring offers a simple, scalable, and cost‐competitive route to thinning Li metal. However, further refinement is imperative to achieve uniform thickness and to suppress the cracks and wrinkles that arise from processing‐induced mechanical stresses. In addition, melt‐processing techniques encounter substantial obstacles to large‐scale production, primarily due to the high reactivity of molten Li and its strong adhesion to molds and processing equipment. By contrast, physical vapor deposition allows precise control over both the thickness and morphological uniformity of ultrathin Li metal. Techniques such as pulsed laser deposition and sputtering, however, are economically limited by low deposition rates and inherent scaling challenges. Notably, thermal evaporation, leveraging lithium's high vapor pressure and low melting point, achieves higher deposition rates while producing films with excellent homogeneity and surface conformality, underscoring its potential for the scalable fabrication of ultrathin Li metal for practical applications.

Additionally, achieving highly reversible Li plating and stripping with high Coulombic efficiency are indispensable for sustaining long‐term operation of ultrathin Li metal, given its intrinsically limited lithium reservoir. For 17 µm Li metal, sustaining 1250 cycles with 75% capacity retention necessitates an average Coulombic efficiency of ≥99.929% [15]. As the thickness is further reduced to 15 µm, achieving even higher Coulombic efficiency becomes imperative. Moreover, in ultrathin Li metal, the accumulation of mechanical stress during cycling, together with dendritic growth, can lead to through‐thickness cracking that fragments the electrode into electrically isolated “dead Li” domains. Such electrode fragmentation typically precedes dendrite short‐circuiting, triggering rapid degradation and potential cell failure. Consequently, preserving the mechanical integrity of ultrathin Li metal throughout repeated cycling is of critical importance. The exacerbated interfacial degradation, the accumulation of impedance arising from thick, resistive solid electrolyte interphase, and crack‐induced mechanical instability collectively underscore the imperative for rational interfacial and structural engineering of ultrathin Li metal to prolong its cycling lifespan (Figure 17).

The formation and stabilization of a robust SEI is fundamentally critical for enabling the stable operation of ultrathin Li metal anodes. From the perspective of electrolyte engineering and interfacial chemistry, this can be achieved through rational regulation of the solvation environment. By tuning cation/anion–solvent interactions, the solvation structure can be engineered to direct the formation of inorganic‐rich SEI layers, thereby suppressing parasitic reactions and minimizing Li loss. Importantly, future efforts should focus on the fundamental understanding and precise regulation of interfacial electron‐transfer processes, as optimized electronic coupling and reduced energy barriers are critical for enabling efficient Li^+^/Li° conversion. This dual control over SEI chemistry and electron transfer is essential for improving plating/stripping reversibility and extending the practical operating window of ultrathin Li metal anodes, particularly under demanding conditions such as wide‐temperature operation and ultrafast charging. Notably, such interfacial regulation should move beyond an anode‐centric perspective. In practical full‐cell configurations, crossover‐coupled interphase chemistry between the cathode and anode, mediated by electrolyte species and their decomposition products, can profoundly influence the formation and evolution of the anode SEI. Therefore, future efforts should focus on the systematic investigation and rational design of these cross‐interface interactions.

Deliberate construction of robust artificial interphase layers to passivate ultrathin Li metal either through direct coating or in situ derivation by additives can effectively stabilize the ultrathin Li metal, mitigating parasitic reactions with the electrolyte. Such interfacial engineering requires the concerted integration of electrochemical insight, mechanical design, and precise process control. And employing solid electrolytes capable of suppressing both dendritic Li growth and Li loss also represents a promising strategy for stabilizing ultrathin Li metal anodes. To ensure structural stability and preserve the mechanical integrity of ultrathin Li metal during cycling, the adoption of three‐dimensional structured current collectors, integrated with lithiophilic nucleation sites, offers a viable strategy to accommodate the substantial volume changes during the repeated Li plating/stripping.

The synergistic integration of mechanical thinning with interfacial and structural engineering is expected to advance the practical viability of ultrathin Li metal, paving the way for next‐generation batteries with high energy density (> 500 kg^−1^) and enhanced safety. Concurrent progress in materials science and manufacturing engineering will be pivotal for enabling the industrial‐scale production of ultrathin Li metal. Furthermore, comprehensive techno‐economic assessments—incorporating sensitivity analyses of lithium carbonate prices, electricity costs, carbon offsets, and labor—are critical to inform and guide large‐scale implementation. Looking ahead, the rigorous evaluation of ultrathin Li metal will necessitate the establishment of quantitative performance metrics and well‐defined benchmarks, including precise thickness control, explicit correlations between areal capacity and cycle life, and the extent of volumetric expansion during operation. Future breakthroughs will likely emerge from the co‐design of electrolyte chemistry, interphase dynamics, and scalable fabrication, thereby transforming ultrathin Li metal from a fragile component into a chemically and mechanically regulated subsystem.

## Funding

This work was supported by the Australian Research Council (FT230100598 and FL210100050). S.X. acknowledges the financial support from the National Natural Science Foundation of China (52371230); Shanghai Pujiang Talent Program (25PJD095); Shanghai Eastern Talent Plan (QNJY2025138); and Science and Technology Commission of Shanghai Municipality (25520750100).

## Conflicts of Interest

The authors declare no conflicts of interest.

## Supporting information




**Supporting File**: adma73568‐sup‐0001‐SuppMat.docx.

## Data Availability

The data that support the findings of this study are available from the corresponding author upon reasonable request.
