# Supplementary material for: Ultrathin Li Metal Anodes: Quantitative Design Principles and Manufacturability Across Liquid and Solid‐State Batteries
Source: Adv Mater. 2026 Jun 1;38(38):e73568. doi: 10.1002/adma.73568 (PMC13351781; doi:10.1002/adma.73568)
Supplement: Supplementary file 1 — Supporting File: adma73568‐sup‐0001‐SuppMat.docx. [file ADMA-38-e73568-s001.docx]

Supporting Information

Ultrathin Li metal anodes: quantitative design principles and manufacturability across liquid and solid-state batteries

*Cheng Wang, Caoyu Wang, Shuixin Xia,^*^ Jodie A. Yuwono, Mingnan Li, Yanqiu Lyu, Ruizhi Zhang, Jun Wang, Jianfeng Mao,^*^ Zaiping Guo^*^*

**Methodology Section: Detailed Explanation of Specific Energy Illustrated in Figure 1**

This section provides a detailed explanation of the specific energy presented in Figure 1, which depicts the variation in specific energy of batteries with different cell parameters when cathodes are paired with lithium anodes of varying thicknesses. The specific energy of the battery—both gravimetric (Eg, in Wh kg^-1^) and volumetric (Ev, in Wh L^-1^)—can be calculated as follows:

$$E_{g}=\frac{C_{cell}\times V_{cell}}{\sum m_{components}}$$

$$E_{v}=\frac{C_{cell}\times V_{cell}}{\sum v_{components}}$$

Where $C_{cell}$ represents the battery capacity (in Ah), $V_{cell}$ denotes the cell voltage (in V), and $m_{components}$ is the total weight of the battery components (in kg). The battery capacity $C_{cell}$ is determined by the specific capacity of the cathode material and the cathode loading, given as $C_{cell}\left( Ah \right)=C_{spcath}\left( Ah kg^{-1} \right)\times m_{cath}\left( kg \right)$. The cell voltage $V_{cell}$ is typically defined as the midpoint voltage during discharge. The total weight of the components ​$\sum m_{components}$ includes all constituents of the battery, such as active materials (cathode material, lithium anode, and electrolyte) and inactive materials (conductive carbon, binders, current collectors, and separators). To achieve a practical lithium metal battery, all cell parameters are configured similarly to those of conventional lithium-ion batteries. Porosity and true density values for various cathodes are estimated based on references. To control weight and achieve high energy density within a limited volume, the cathodes are double-sided coated, with a single-side loading of 5 mAh cm^-2^. The lithium metal anode uses a 6 µm-thick copper foil as the current collector, while the cathode current collector is a 10 µm-thick copper foil. Each pouch cell contains 20 double-sided cathode layers and 19 double-sided plus 2 single-sided anode layers. Considering the high reactivity of lithium metal and electrolyte, along with the porous lithium formed during deposition and redeposition, the electrolyte content is set at 3.0 g (Ah)^-1^.

For the energy density calculation of all-solid-state lithium metal batteries (SSBs), we employed the Solid-State Battery Performance Analyzer and Calculator (SolidPAC), an experimental and interactive SSB design toolkit developed by Oak Ridge National Laboratory. A key feature of SSBs is their ability to utilize bipolar stacking. This design enhances the battery's rated voltage while minimizing inactive elements (e.g., packaging and electrical connections), thereby improving energy density. Consequently, the EV battery model is configured with a bipolar stack for evaluation.

In this model, the thickness of the solid-state electrolyte is set to 100 µm, and the cathode areal capacity is maintained at 5 mAh cm^-2^. Additional parameters are detailed in **Table S1**.

**Table S1**. Parameters for ASSLBs energy density calculation

| **Cathode Design** | | **Current Collecter Design** | |
| --- | --- | --- | --- |
| Active material fraction | 80 | -Ve CC thickness (μm) | 10 |
| Catholyte fraction | 10 | +Ve CC thickness (μm) | 15 |
| Carbon fraction | 5 |  |  |
| Binder fraction (%) | 5 |  |  |
| Cathode porosity (%) | 15 |  |  |
| Cathode areal loading (mAh cm^-2^) | 5 |  |  |
| **Cell Design Parameters** | | **Battery Design Parameters** | |
| Battery Pack Insulation Thickness (mm) | 10 | Number of Packs/Vehicle | 1 |
| Thickness of cell container Al layer (μm) | 100 | Cells/module | 12 |
| Lenght to Width Ratio for Cathode | 3 | Cells in parallel | 1 |
| Cell Thickness Target (mm) | 20 | Modules in a row | 5 |
| Thickness of cell edge from cathode to outside of fold (mm) | 1 | Rows of modules/pack | 4 |
| Thickness of terminal material (mm) | 1 | Modules in parallel Energy | 1 |
| Distance of top of cathode to the top of terminal (mm) | 15 | Requirement (Wh/mile) | 250 |
|  |  | Formation Cycles Capacity Loss (%) | 10 |
